# Heat shock protein 90-targeted photodynamic therapy enables treatment of subcutaneous and visceral tumors

**DOI:** 10.1038/s42003-020-0956-7

**Published:** 2020-05-08

**Authors:** Kensuke Kaneko, Takuya Osada, Michael A. Morse, William R. Gwin, Joshua D. Ginzel, Joshua C. Snyder, Xiao-Yi Yang, Cong-Xiao Liu, Márcio A. Diniz, Khaldon Bodoor, Philip F. Hughes, Timothy AJ. Haystead, H. Kim Lyerly

**Affiliations:** 1 Department of Surgery, Duke University Medical Center, Durham, NC 27710 USA; 2 Department of Medicine, Duke University Medical Center, Durham, NC 27710 USA; 3 Department of Medicine, University of Washington, Seattle, WA 98195 USA; 4 Department of Cell Biology, Duke University, Durham, NC 27710 USA; 5 Biostatistics and Bioinformatics Research Center, Samuel Oschin Comprehensive Cancer Institute, Cedars-Sinai Medical Center, Los Angeles, CA 90048 USA; 6 Department of Pharmacology and Cancer Biology, Duke University, Durham, NC 27710 USA

**Keywords:** Breast cancer, Targeted therapies

## Abstract

Photodynamic therapy (PDT) ablates malignancies by applying focused near-infrared (nIR) light onto a lesion of interest after systemic administration of a photosensitizer (PS); however, the accumulation of existing PS is not tumor-exclusive. We developed a tumor-localizing strategy for PDT, exploiting the high expression of heat shock protein 90 (Hsp90) in cancer cells to retain high concentrations of PS by tethering a small molecule Hsp90 inhibitor to a PS (verteporfin, VP) to create an Hsp90-targeted PS (HS201). HS201 accumulates to a greater extent than VP in breast cancer cells both in vitro and in vivo, resulting in increased treatment efficacy of HS201-PDT in various human breast cancer xenografts regardless of molecular and clinical subtypes. The therapeutic index achieved with Hsp90-targeted PDT would permit treatment not only of localized tumors, but also more diffusely infiltrating processes such as inflammatory breast cancer.

## Abstract (web summary)

Heat shock protein 90 (Hsp90) is highly expressed in cancer cells. Kaneko et al show that targeting Hsp90 on the cancer cell surface with a photosensitizer tethered to an Hsp90 small molecule inhibitor improves the efficacy of photodynamic therapy in various human breast cancer xenografts, suggesting a therapeutic avenue for human disease.

## Introduction

Photodynamic therapy (PDT) of cancer involves administering a photoactive compound that when taken up by tissues and exposed to specific wavelengths of light, leads to conversion of oxygen to reactive oxygen species, resulting in direct cytotoxic effects on tumor cells, occlusion of the tumor vasculature, and inflammatory and adaptive immune responses^1–4^. Although exhibiting some preferential accumulation in tumor tissue, photosensitizers (PS) are not tumor-exclusive. Consequently, therapeutic ablation of tumor is achieved by the targeted delivery of light to the PS, however, PS within the non-tumor tissue will also be activated when exposed to environmental light, resulting in phototoxicity. Clinically available PS also persist in normal tissue for substantial periods necessitating prolonged protection from light. For example, the FDA-approved PS, porfimer sodium (Photofrin) has a half-life of 21.5 days, necessitating avoidance of sunlight exposure (which can activate it) for approximately 6 weeks after treatment to avoid skin toxicities. Second-generation synthetic PS, such as temoporfin and verteporfin (VP) have shorter half-lives to address these concerns.^5^

VP is currently approved by the FDA for the treatment of age-related macular degeneration^6^, but it is of particular interest for PDT of solid tumors, because (1) peak tissue concentration occurs within an hour or two after systemic administration^7,8^, (2) rapid clearance (by excretion in bile) reduces the window of risk for skin photosensitivity to 24 h^9^, (3) it has an increased absorption at 690 nm, a wavelength at which light penetrates tissue deeper than other PS with shorter red wavelength absorption peaks. PDT using VP (VP-PDT) along with a local light source (such as a percutaneous light source) has been demonstrated to ablate prostate and pancreatic tumors in preclinical cancer models^10,11^. A phase I/II clinical trial demonstrated that VP-PDT was safe and effective at inducing tumor necrosis in locally advanced pancreatic cancers^12^. Nonetheless, treatment of deeper tumors with VP-PDT depends on the percutaneous delivery of laser fibers into tumors and laser illumination from inside the tumors, rather than the selective uptake of the PS into the tumor tissue.

To overcome this barrier, and enable PDT for visceral or diffuse superficial tumors, we developed a strategy for selective tumor targeting of the PS by exploiting molecular signaling unique to cancer, specifically heat shock protein 90 (Hsp90). Hsp90 is a well-known chaperone protein that is highly expressed in a variety of malignancies where it is associated with more aggressive growth and worse outcome^13–15^. In breast cancer (BC), elevated Hsp90 was found to be associated with a higher rate of disease recurrence and a poor prognosis^13,16^. Cell surface expression of Hsp90 was reported in malignancies^17,18^, including BC^19–21^, and thus Hsp90 can be a widely-applicable target of the PS in tumor-targeting PDT. Hsp90 inhibitors have been found to accumulate in cancers to high levels in vivo^22^. We recently demonstrated an Hsp90 small molecule inhibitor could be tethered to a near infrared (nIR) probe (HS131), and would specifically accumulate in all molecular subtypes of BC in vitro and in vivo^20^. High resolution confocal microscopy has shown that probe-bound Hsp90 accumulates in punctate structures on the plasma membrane and was actively internalized into the cytosol of breast tumor cells^21^. HS131 was preferentially retained for up to 72 h by cancer cells, but rapidly left normal tissues and organs. To exploit these properties, we chemically tethered VP to the same small molecule Hsp90 inhibitor (HS10) to create an Hsp90-targeted PS (HS201) for use with PDT.

Because HS201 is expected to specifically label all molecular subtypes of BCs, consistent with our observation with our imaging probe HS131, we sought to assess the specific accumulation of HS201 in tumor cells in vitro and in vivo, and evaluated the antitumor efficacy of HS201-based PDT (HS201-PDT) across all BC subtypes, including inflammatory BC (IBC) and subtypes that recur in the chest wall (where PDT is currently used^23,24^). Indeed, targetable Hsp90 is found in IBC cell lines which are highly susceptible to Hsp90 inhibition^25^ and therefore, we hypothesized that this clinical BC subtype would be particularly appropriate for HS201-based therapy. We also expect this strategy to enable cancer-specific treatment of deeper lesions, without the need for invasive light sources, while also minimizing phototoxicity.

## Results

### Higher and prolonged uptake of HS201 in BC cells in vitro

**Fig. 1:**
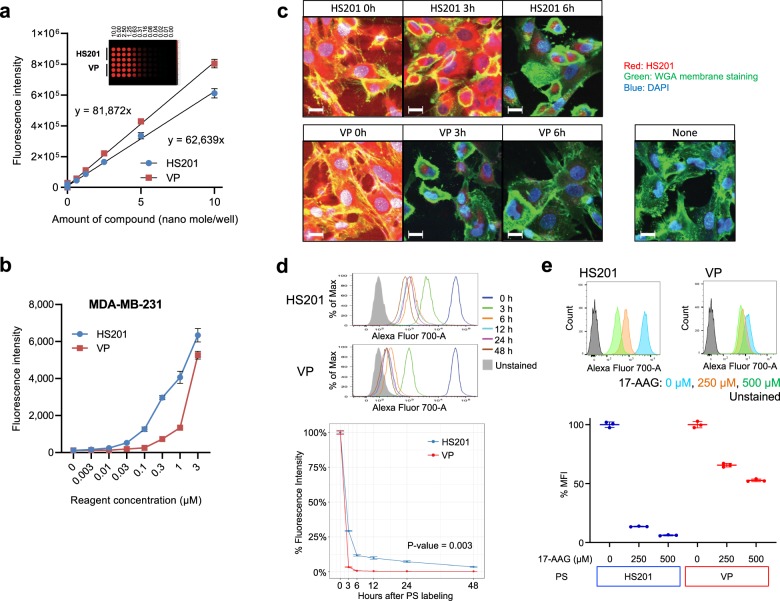
Uptake of the two PS, HS201, and VP, by human BC cells in vitro. **a** nIR signal at 700 nm wavelength emitted from HS201 and VP. Indicated amount of PS (HS201 or VP) was added to each well and nIR signal intensity was measured at 700 nm. Standard curves for both PS are shown. Data are expressed as means ± SD. **b** Uptake of HS201 and VP by human BC cells in vitro. MDA-MB-231 cells were labeled with HS201 or VP (0–3 μM, respectively). nIR fluorescence intensity of each well, measured at 700 nm are shown as means ± SD. **c** Confocal microscope images of MDA-MB-231 cells labeled with HS201 and VP (1 μM) in vitro. Cells were fixed immediately after nIR staining (0 h group), or were washed with DMEM every hour until designated time point and fixed (3 and 6 h groups). Then the cells were stained with WGA AF488 conjugate membrane staining dye and DAPI, and observed by a ZEISS LSM880 confocal microscope. Original magnification: Objective 63×. The scale bar indicates 20 μm. **d** Flow cytometry analysis of nIR signal accumulated in MDA-MB-231 cells. Cells were labeled with HS201 or VP (1 μM), and then fixed immediately (0 h group) or washed with DMEM every 1 h until 12-h time point (3, 6, and 12 h groups) and every 12 h thereafter (24 and 48 h groups). The histogram shows the signal intensity of representative samples at each time point. The graph shows percentages of mean fluorescence intensities (MFI) when compared with the samples of 0 h group (100%). All experiments were performed in triplicate and data are shown as means ± SEM. Student’s *t* test was performed for the comparison of %MFI. **e** Uptake of PSs by MDA-MB-231 cells in the presence or absence of 17-AAG in vitro. The histogram shows the nIR signal intensity of representative samples at each condition. MFI of cells in the absence of 17-AAG were set as 100% for each PS, and MFI of each condition is shown as %MFI. *N* = 3 for each condition. Data are expressed as means ± SD.

We first compared the fluorescence signal of the two compounds, HS201 and VP, when exposed to a laser of 690 nm wavelength using the LI-COR Odyssey Imager (Fig. 1a). VP showed approximately 1.3 times stronger nIR signal than HS201 at the same concentrations when diluted in DMSO.

We compared the binding activity of HS-10, an Hsp90 inhibitor, HS201, and HS205, an inactive analog of HS201. We used two methods: (1) the amount of a recombinant Hsp90–GFP fusion protein eluted from an ATP resin by the HS201 and its analogs, (2) ELISA using purified human Hsp90 protein. As demonstrated in Supplementary Fig. 1a–d, we confirmed that HS10 and HS201 bind human Hsp90 while HS205 and VP do not.

Next, we tested the cell surface expression of Hsp90 protein on BC cells by flow cytometry and confocal fluorescence microscopy (Supplementary Fig. 2). MCF7, BT474M1, KPL-4, and MDA-MB-231 cells were shown to have mild expression of Hsp90 on the cell surface based on the flow cytometry analysis using alive non-permeabilized cells. Focal expression of Hsp90 on the plasma membrane was demonstrated for MDA-MB-231 cells by confocal fluorescence microscopy. Then, the in vitro uptake of HS201 and VP into the BC cells was tested. HS201 uptake statistically exceeded what was observed with VP in MDA-MB-231 cells when the reagent concentration was less than 3 µM (Fig. 1b). A similar trend was observed in other human BC cell lines: MCF7, KPL-4, and BT474M1 cells (Supplementary Fig. 3a). Importantly, normal mammary epithelial cell lines such as MCF10A and HMEC showed significantly higher uptake of VP compared to HS201 even at lower concentrations of these reagents. These findings suggest that BC cells may have a specific mechanism to effectively take up HS201 which does not function for the uptake of VP. To confirm that this mechanism was in part Hsp90-mediated, we also tested an inactive version of HS201 (HS205) which cannot bind Hsp90 protein. We demonstrated that the accumulation of HS201 exceeded that of HS205 in MDA-MB-231 cells (Supplementary Fig. 3b).

Next, we confirmed the intracellular localization of HS201 and VP by confocal microscopy (Fig. 1c). MDA-MB-231 cells were co-incubated with HS201 or VP for 30 min and washed. The cells of the 0 h group were fixed and stained with DAPI and WGA Alexa Fluor 488 immediately. The cells of the 3- and 6-h groups were washed with fresh media every hour, fixed and stained with DAPI and WGA Alexa Fluor 488 at each designated time point. The localization of nIR signals on the cell membrane and in the cytoplasm was observed soon after the incubation, suggesting the binding of these compounds to the cell membranes and incorporation into the cytoplasm. Importantly, the nIR signal of VP was detectable in the cytoplasm for a shorter time frame and had diminished from the cells by the 6-h time point, while HS201 showed longer retention in the cytoplasm. We also generated 3D images of tumor cells stained 6 h after HS201 co-incubation, which demonstrates that HS201 (pink) was incorporated into the cytoplasm (Supplementary Movie 1).

To confirm the findings of the confocal microscope analysis, the retention of HS201 and VP in MDA-MB-231 cells was evaluated using flow cytometry after incubation for up to 48 h (Fig. 1d). Beginning at 3 h after the cellular uptake of PSs, HS201 demonstrated a signal retention that was more than ten times stronger than was noted with the VP.

We also performed HS201 uptake analysis in the presence of 17-AAG, an Hsp90 inhibitor, to further demonstrate Hsp90 specific uptake (Fig. 1e). HS201 uptake was reduced to less than 15% with 250 μM of 17-AAG and to approximately 6% with 500 μM 17-AAG, while VP uptake was maintained more than 50% at the same doses.

Because tumor cell uptake of VP through LDL receptors was reported previously^26^, the uptake of HS201 and VP by MDA-MB-231 cells following LDL receptor blockade was also tested to assess the role of LDL receptors in HS201 uptake (Supplementary Fig. 4). VP uptake in MDA-MB-231 cells was impaired when the cells were pre-incubated with LDL (5, 10, and 25 µM) while the effect on HS201 uptake was significantly smaller in the same conditions. These results suggest the existence of a cancer-specific mechanism for the uptake of HS201 by cancer cells; however, LDL receptors play a minor role in the uptake of HS201 by BC cells and therefore other mechanisms must be operative.

### In vitro HS201-PDT surpasses VP-PDT in killing tumor cells

**Fig. 2:**
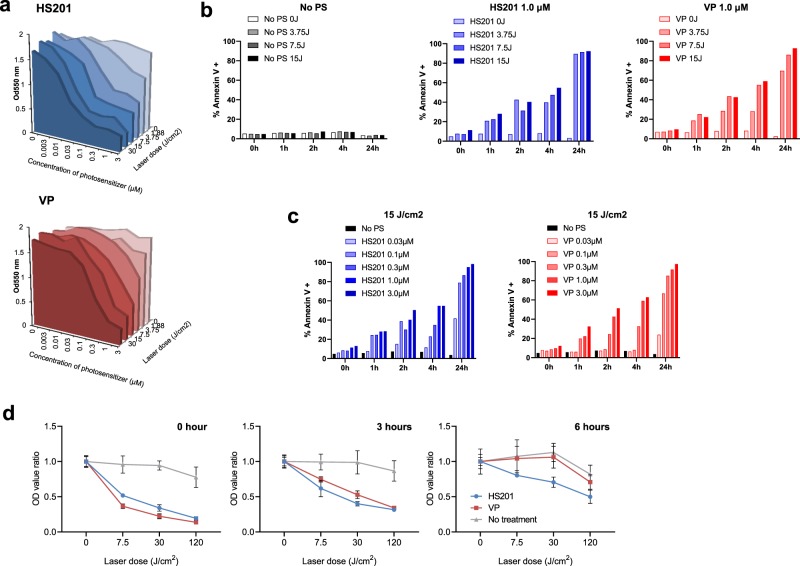
Killing of human BC cells by in vitro PDT with HS201 and VP. **a** Cytotoxicity of in vitro HS201-PDT and VP-PDT analyzed by MTT assay. MDA-MB-231 cells seeded in 96-well plates were labeled with HS201 or VP (0–3 μM), irradiated by 690 nm wavelength laser (0–30 J/cm^2^), cultured overnight and analyzed by MTT assay. Upper graph shows the result for HS201-labeled cells, and lower graph shows VP-labeled cells. **b** Flow-based analysis of apoptotic cell death induced by HS201-PDT and VP-PDT with fixed PS dose (1 μM) and titrated laser dose (0–15 J/cm^2^). Cells were incubated for 0–2 and 4 h or overnight at 37 °C after treatment, labeled with Annexin V and 7-AAD, and acquired by an LSRII flow cytometer. Percentages of Annexin V positive cells at each time point are shown. **c** Flow-based analysis of apoptotic cell death induced by HS201-PDT and VP-PDT with fixed laser dose (15 J/cm^2^) and titrated PS dose (0.03–3 μM). Cells were labeled, acquired and analyzed as described in (**b**). **d** Drug-light interval and the killing effect of in vitro PDT. MDA-MB-231 cells were labeled with HS201 or VP (1 µM), washed every hour, and irradiated with laser (0–120 J/cm^2^) at each time point (0, 3, or 6 h). Cells were incubated overnight and MTT assay was performed. OD ratio is shown as a fold change when compared with cells untreated with laser. Data are expressed as means ± SD.

Cytotoxicity induced by in vitro PDT was analyzed by an MTT assay after overnight incubation of PDT-treated cells. Using the MDA-MB-231 cells immediately after the incubation with HS201 or VP, we confirmed that the killing effect of PDT was dependent on both laser and reagent dose (Fig. 2a). In addition, we tested other human BC cell lines such as MCF7, KPL-4, and BT474M1 and normal mammary epithelial cell lines such as MCF10A and HMEC cells (Supplementary Fig. 5a). Human BC cells were more sensitive to HS201-PDT than VP-PDT, whereas normal mammary epithelial cell lines showed the opposite tendency. These different killing efficacies between BC cells and normal mammary epithelial cells corresponded to the level of cellular uptake of HS201 and VP. We also tested the inactive analog HS205 in in vitro PDT and found that killing efficacy of HS205-PDT was lower than that of HS201-PDT and was similar to that of VP-PDT, again corresponded to the level of cellular uptake of PS (Supplementary Fig. 5b).

To elucidate the mechanism of cell killing by HS201-PDT and VP-PDT, flow-based apoptosis assays were performed using MDA-MB-231 cells. First, MDA-MB-231 cells were incubated with HS201 or VP (1 μM) followed by titrated laser ablation (0–15 J/cm^2^), incubated for each designated time (0–24 h), and then stained with Annexin V and 7-AAD. Both PDTs (VP-PDT and HS201-PDT) induced apoptotic cell death of MDA-MB-231 cells in a laser dose-dependent manner. HS201-PDT showed a slightly higher killing effect at a lower laser dose compared to VP-PDT, probably because of higher uptake of HS201 by cancer cells (Fig. 2b). We also tested different PS concentrations (0.03–3 μM) accompanied by a 15 J/cm^2^ laser dose, which similarly resulted in apoptotic cell death of MDA-MB-231 cells in a PS dose-dependent manner (Fig. 2c).

Next, we performed laser exposure to cells immediately (0), 3, or 6 h after initial incubation with PS (1 μM, Fig. 2d). The killing efficacy of PDT was dependent on both laser, reagent dose, and PDT used. MDA-MB-231 cells showed higher sensitivity to HS201-PDT compared to VP-PDT at the 3- and 6-h time points. In particular, VP-PDT showed no effect at the 6-h time point, which corresponded to the diminished intracellular VP (Fig. 1c).

### HS201 shows higher accumulation than VP in the tumor in vivo

**Fig. 3:**
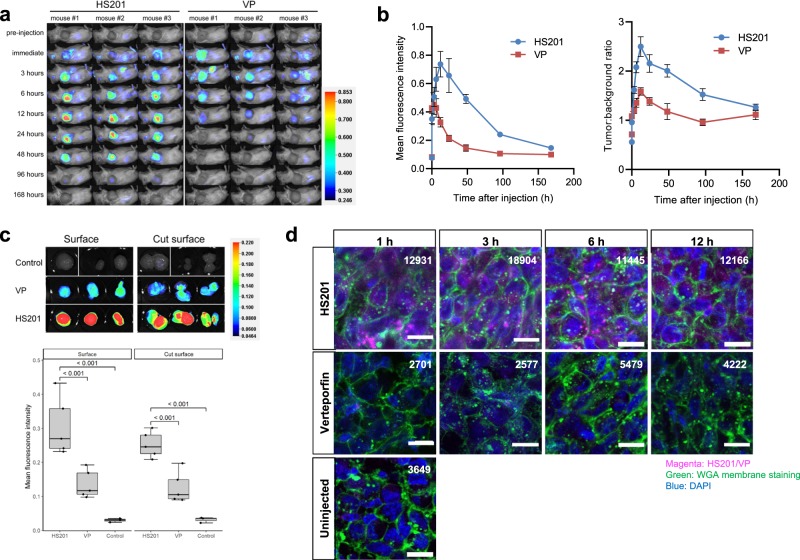
Temporal dynamics of HS201 and VP distribution in human BC xenografts-bearing mice. **a** nIR signal from MDA-MB-231 tumor-bearing mice injected with HS201 or VP. HS201 or VP (10 nmol) were administered via tail vein, and nIR signals from tumor areas were detected by the LI-COR Pearl imager at 700 nm channel over time (pre-injection, immediate, 3, 6, 12, 24, 48, 96, and 168 h after injection). **b** Temporal dynamics of nIR signal from MDA-MB-231 tumors by in vivo imaging. Fluorescence intensities were monitored for individual tumors over time by Pearl Imager and average values (*n* = 5 mice) are plotted. Half-life of the nIR signal in the tumor site for HS201 and VP was 50 and 13 h, respectively. The ratios of nIR signals detected at the tumor site and background skin around the ear were also calculated and the average values (*n* = 5 mice) were plotted. Data are expressed as means ± SEM. **c** Ex vivo imaging of MDA-MB-231 tumors. Mice administered 10 nmol of HS201 or VP were sacrificed at the 24 h time point (5 mice for each VP and HS201 group, 3 mice for control group) to harvest the tumors. nIR signals from excised tumors and their cut surfaces were measured using the Pearl Imager at the 700 nm channel. Three representative tumors from each group are shown. In the two graphs, mean nIR signal intensities of the tumor surface and cut surface are shown. Kruskal–Wallis test was performed followed by non-parametric Dunnett multiple comparisons. **d** Confocal microscope images of excised MDA-MB-231 tumors. MDA-MB-231 tumors were harvested 1, 3, 6 or 12 h after the PS injection. Tumors were fixed overnight in formalin before sectioning. Samples were stained with WGA Alexa Fluor 488 conjugate membrane staining dye and DAPI and observed using a ZEISS LSM880 confocal microscope. nIR signals from photosensitizers are shown in magenta color. Mean fluorescence intensities of nIR signals for tile scanned large tumor sections are shown in each microscope image. Original magnification: Objective 40×. The scale bar indicates 15 μm.

The temporal dynamics of nIR signal as evaluated by the tumor area in mice injected with HS201 or VP is shown in Fig. 3a, b. The ratios of nIR signal detected at the tumor site and background skin around the ear were also calculated and plotted in the graph as the tumor:background ratio. Peak levels of nIR signals at the tumor site and the tumor:background ratio were significantly higher in HS201-injected mice than in the VP-injected mice. In addition, the higher levels of nIR signal and tumor:background ratio were observed for a longer period of time in HS201-injected mice, suggesting the longer retention of HS201 inside the tumors compared to VP. The half-life of each compound at the tumor site was calculated taking each peak time point as a starting point. The half-life of VP at the tumor site was approximately 13 h while HS201 was as long as 50 h. To confirm that this mechanism was in part Hsp90-mediated, we demonstrated that the accumulation of HS201 exceeded that of HS205, an analog without Hsp90 binding, in MDA-MB-231 tumors in mice (Supplementary Fig. 6a, b).

To confirm the in vivo uptake and longer retention of HS201 in MDA-MB-231 tumors, mice were sacrificed at the 24-h time point and nIR signals from the tumor surface and cut surface were analyzed (Fig. 3c). Increased accumulation of HS201 compared to VP was observed based on both surface and cut surface imaging. Importantly, wide distribution of HS201 inside the tumors was confirmed from the nIR images of cut surfaces, which would enable efficient tumor ablation with laser irradiation. In addition, we compared the uptake of PS by tumor cells after the injection of HS201 and VP (1, 3, 6, and 12 h) by confocal microscope analysis (Fig. 3d). The images of tile scanned large area of tumor sections are shown in Supplementary Fig. 7. The mean fluorescence intensities of the tumor tissues were calculated for the whole tumor area of the sections and shown in each microscope image in Fig. 3d. The nIR signal showed that HS201 had stronger accumulation in the tumor cells compared to VP. Dotted and diffuse distribution of nIR signals detected in the cytoplasm of tumor cells suggested that significant amounts of HS201 were internalized and retained inside the tumor cells at least until 12 h time point after intravenous administration. We also analyzed the temporal dynamics of nIR signal level in the plasma to evaluate the plasma half-life of HS201 and VP (Supplementary Fig. 8a). First, fluorescence signals of HS201 and VP were compared when these two compounds were diluted in murine blood plasma, measured by the LI-COR Odyssey Imager as shown previously (Fig. 1a). VP showed approximately 1.3 times stronger nIR signal than HS201 at the same concentrations, which is compatible with the former result (Supplementary Fig. 8b). The temporal dynamics of two PSs demonstrated that VP was eliminated from the peripheral blood more rapidly compared to HS201 (Supplementary Fig. 8c). The half-life of VP and HS201 in plasma was approximately 1.7 and 3.3 h, respectively, while it took 13 and 50 h, respectively, to halve the nIR signal in tumor area. These results suggest that HS201 has a relatively short half-life period in peripheral blood while it is retained longer in the tumor tissue.

**Fig. 4:**
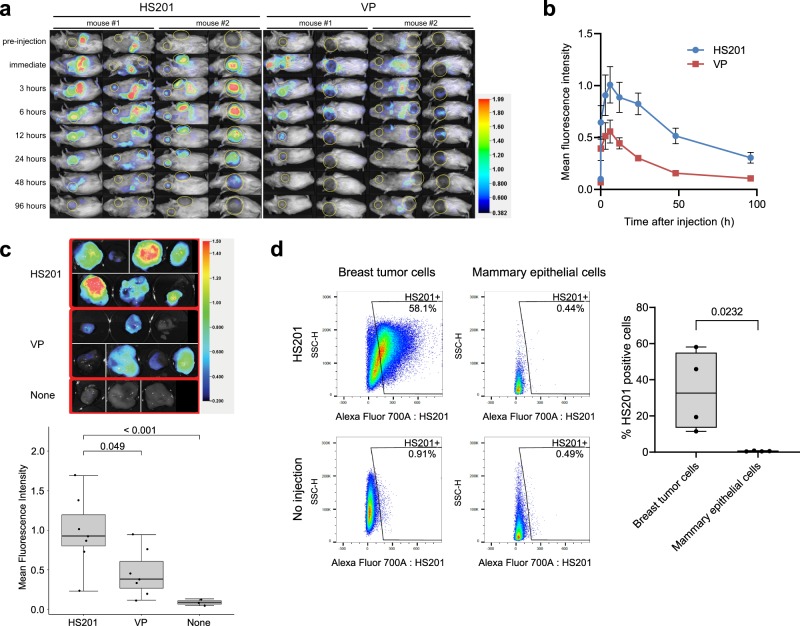
Uptake of HS201 and VP by murine spontaneous BC. **a** nIR signal from breast tumor-bearing MMTV-neu mice injected with HS201 or VP. MMTV-neu mice that developed spontaneous BCs with the size of 5–15 mm in diameter were selected, and administered 25 nmol of HS201 or VP via tail vein. nIR signals from the tumor area were detected using a Pearl Imager over time. The images of two representative mice from each group are shown. **b** Temporal dynamics of nIR signal intensity from spontaneous tumors by in vivo imaging. The average fluorescence intensities (5 mice for HS201 group and 6 mice for VP group) are shown. Half-life of HS201 and VP signal detected in the tumor site was 42 and 15 h, respectively. Data are expressed as means ± SEM. **c** Ex vivo imaging of spontaneous tumors in MMTV-neu mice. Mice (5 mice 7 tumors for HS201 group, 4 mice 7 tumors for VP group, and 2 mice 3 tumors for control group) were sacrificed at the 6-h time point after HS201 or VP (25 nmol/mouse) injection. nIR signals from excised tumors are shown in the graph. Kruskal–Wallis test was performed followed by nonparametric Dunnett multiple comparisons. **d** Flow cytometry analysis of HS201 uptake by breast tumor cells and normal mammary epithelial cells in tumor-bearing MMTV-neu mice. HS201 (100 nmol/mouse) was injected into MMTV-neu mice (three tumor-bearing mice with four tumors and two non tumor-bearing mice) via tail vein. Control mice (one tumor-bearing mouse and one non tumor-bearing mouse) received no compound injection. Mice were sacrificed 6 h after compound injection. Tumors and mammary gland tissues were digested into single cells, acquired by an LSRII flow cytometer and nIR signals from tumor cells and mammary epithelial cells (CD24 positive and CD45 negative cells) were analyzed. Dot plots of tumor and epithelial cells from a representative mouse are shown with percentages of HS201 positive cells. Percentages of HS201-positive cells in four samples of breast tumor cells and four samples of normal mammary epithelial cells are shown as a box plot, and *t* test was performed.

Similarly, we evaluated the uptake in a spontaneous BC model (MMTV-neu mice) more relevant to human tumors (Fig. 4a, b). The nIR signal accumulation in the tumors was significantly higher in HS201 injected mice than VP and no PS injection treatment conditions (HS201 vs. VP *p* = 0.049, HS201 vs. None *p* < 0.001, respectively, Fig. 4c). Moreover, to assess selective uptake of HS201 by tumor cells in vivo, we compared the nIR signals from breast tumor cells and mammary epithelial cells isolated from tumor-bearing MMTV-neu mice that were administered HS201. Specifically, the mammary gland tissues and spontaneous tumors were harvested from mice 6 h after HS201 (100 nmol/mouse) injection, enzymatically digested into single breast tumor cells or mammary epithelial cells, stained with CD24 and CD45, and analyzed by an LSRII flow cytometer (Fig. 4d). nIR signals at the wavelength of 700 nm from tumor cells and mammary epithelial cells (CD24-positive and CD45-negative cells) were measured. Tumor cells had significantly higher population of nIR-positive (uptake of HS201) cells, while mammary epithelial cells were almost completely negative (Fig. 4d). These results demonstrate the tumor selectivity of HS201 in vivo.

To optimize the dose of HS201 for PDT against BCs, we compared in vivo tumor accumulation and tissue distribution of HS201 after the administration of different doses of HS201 (1, 10, 25, 50, and 100 nmol/mouse). Temporal dynamics of PS uptake in a representative mouse from each dosage group are shown in Supplementary Fig. 9a. Signal accumulation peaked at 12 h when HS201 was administrated with the dose of 100 or 50 nmol/mouse, while the peak was 6 h for 25 or 10 nmol/mouse (Supplementary Fig. 9b). The group injected with 25 nmol of HS201 had the highest tumor:background ratio at the majority of time points through 24 h. The mice were sacrificed at the 24-h time point, with subsequent harvesting of tumors and organs. The nIR signal intensity of the harvested tumor increased according to the dosage of HS201 (Supplementary Fig. 9c). We wished to optimize the tumor to normal tissue uptake ratio and found that this occurred at 25 nmol/mouse (Supplementary Fig. 9d). Higher doses led to increased background signal. This result suggests that 25 nmol/mouse would be the optimal dose for HS201 administration to treat a tumor effectively and to avoid healthy tissue damage at the same time.

### HS201-PDT upregulates Hsp90 but inhibits its function

**Fig. 5:**
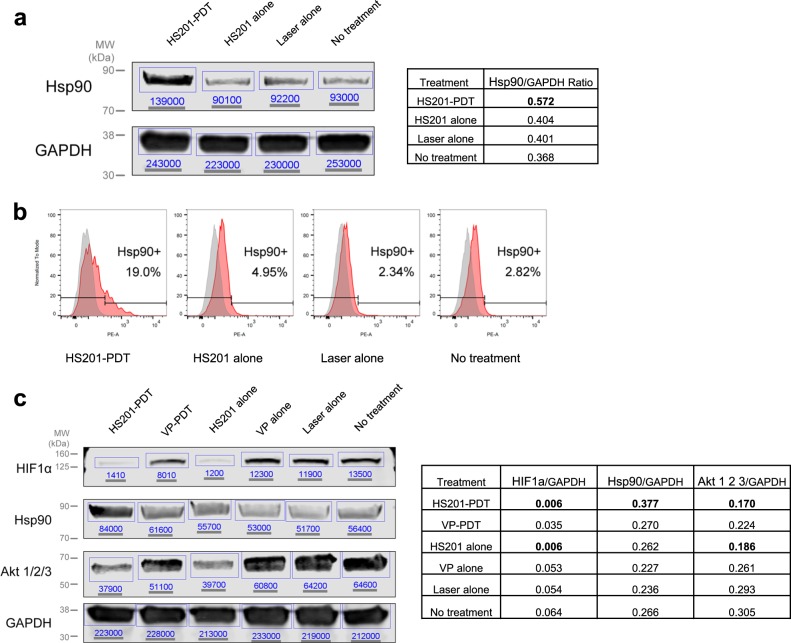
HS201-PDT-induced Hsp90 expression and down regulation of client proteins in human BC cells in vitro. **a** Hsp90 expression in MDA-MB-231 cells treated with or without HS201-PDT in vitro evaluated by Western blot analysis. MDA-MB-231 cells were separated into four groups, HS201-PDT, HS201 alone, Laser alone, and no treatment groups, and treated accordingly. Hsp90 and GAPDH expression in each group were quantified using an Odyssey CLx imaging system. The table shows Hsp90/GAPDH ratio of each group. **b** Surface Hsp90 expression of MDA-MB-231 cells treated with or without HS201-PDT in vitro. MDA-MB-231 cells were treated in the same way as in (**a**). Cell suspensions were prepared and stained with PE-conjugated control IgG or anti-Hsp90 antibody. Surface Hsp90 expression of MDA-MB-231 cells in each group was analyzed by a LSRII flow cytometer. Gray histograms show the cell labeling with control IgG, and the red histograms show the cell labeling with anti-Hsp90 antibody. **c** Expression of Hsp90 client proteins in MDA-MB-231 cells treated with HS201-PDT. MDA-MB-231 cells were treated with HS201-PDT, VP-PDT, HS201 alone, VP alone, Laser alone, or no treatment. HIF1α, Hsp90, Akt 1/2/3, and GAPDH expression in each group were quantified by an Odyssey CLx imaging system. The table shows the ratio of HIF1α, Hsp90, and Akt 1/2/3 to GAPDH, respectively. The images of full-length blots are available in Supplementary Fig. 13.

As HS201 is a compound consisting of VP and an Hsp90 inhibitor, we sought to determine the influence of HS201 administration and HS201-PDT on cellular expression of Hsp90 proteins in tumor cells in vitro and in vivo. First, we compared the Hsp90 expression of cells (by Western blot) after treatment with HS201-PDT (HS201 1 μM, laser 2 J/cm^2^), HS201 alone (1 μM), laser alone (2 J/cm^2^), and no treatment (Fig. 5a). Only the cells treated with HS201-PDT showed upregulation of Hsp90 expression while HS201 or laser exposure alone had no effect. We also compared surface Hsp90 expression on the cells by flow cytometry analysis and observed a similar result that only HS201-PDT treated cells demonstrated increased Hsp90 expression on the cell surface (Fig. 5b). These data indicate that HS201-PDT induces a stress response within treated cells leading to the upregulation of Hsp90.

One concern with increasing Hsp90 expression is the subsequent increase in its capacity to carry its client proteins^27,28^. Accordingly, we evaluated the effect of VP-PDT and VP-alone on the expression of Hsp90 client proteins by Western blotting (Fig. 5c). While upregulated Hsp90 expression was observed only in HS201-PDT treated cells, down regulation of Hsp90 client proteins HIF1α and Akt 1/2/3 protein was confirmed in both HS201-PDT and HS201 alone treated cells.

### Subsequent HS201 accumulation is augmented by laser exposure

**Fig. 6:**
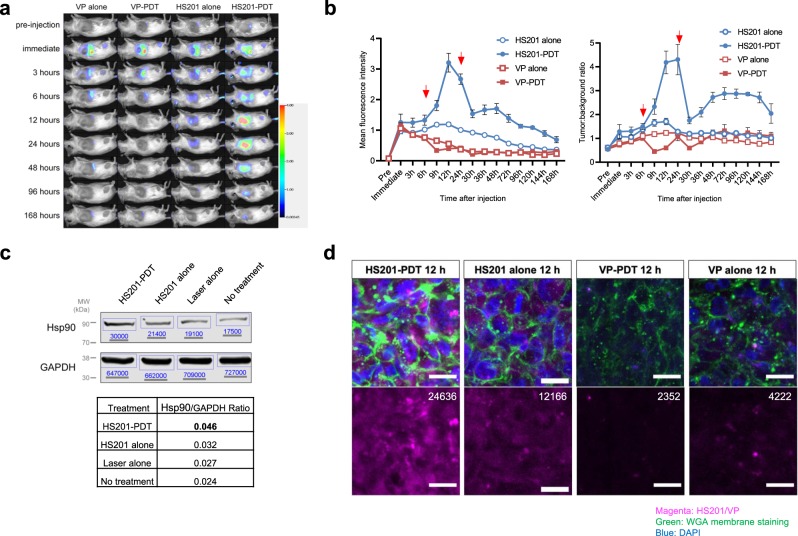
Enhanced HS201 accumulation and Hsp90 expression in human BC xenografts after HS201-PDT in vivo. **a** Enhanced HS201 accumulation in MDA-MB-231 tumors in vivo after laser exposure. MDA-MB-231 tumor-bearing mice were administered HS201 or VP (25 nmol). The laser irradiation (120 J/cm^2^/4 min) was applied twice (drug-light interval of 6 and 24 h) at 690 nm wavelength to the mice in HS201-PDT and VP-PDT groups. Whole-body images were acquired by a Pearl Trilogy imaging system at the 700 nm channel overtime and images of representative mice for each group are shown. **b** Temporal dynamics of nIR signal and tumor:background ratio from MDA-MB-231 tumors by in vivo imaging. The nIR signals detected at the tumor site and shaved and depilated skin around the right thigh were measured. The tumor:background ratio was calculated and plotted in the graph (right panel). Averages ± SEM of nIR signal intensities acquired from five mice per group are plotted in the graph. Red arrows indicate the timing of laser irradiation. **c** Hsp90 expression in MDA-MB-231 tumors treated with or without HS201-PDT in vivo. MDA-MB-231 tumor-bearing mice were administered HS201 (25 nmol/mouse), irradiated with laser (690 nm, 120 J/cm^2^/4 min) 6 h after PS injection, and sacrificed at 12-h time point to collect the tumor lysates. Hsp90 and GAPDH expression by tumors of each group was quantified by Western blot. The table shows Hsp90/GAPDH ratio of each group. **d** Confocal microscope images of excised MDA-MB-231 tumors treated with or without HS201-PDT. MDA-MB-231 tumor-bearing mice were administered with HS201 or VP, followed with or without laser irradiation (690 nm, 120 J/cm^2^/4 min) 6 h after injection, and sacrificed at 12-h time point. Tumors were fixed overnight in formalin before sectioning. Tissue sections were stained with WGA Alexa Fluor 488 conjugate membrane staining dye and DAPI and observed using a ZEISS LSM880 confocal microscope. nIR signals from photosensitizers (magenta color) are shown at the bottom. Mean fluorescence intensities for tile scanned large tumor sections are shown in each microscope image. Original magnification: Objective 40×. The scale bar indicates 15 μm. The images of full-length blots are available in Supplementary Fig. 13.

Having observed increased Hsp90 expression after HS201-PDT, we wished to determine whether this led to increased accumulation of HS201 in the tumor. Mice bearing MDA-MB-231 tumors were imaged following administration of HS201 or VP with or without laser exposure. HS201 accumulates in the tumor site to a greater extent than VP and this effect is markedly increased with the addition of laser exposure. (Fig. 6a). Figure 6b shows the temporal dynamics of the nIR signal accumulation at the tumor site. The nIR signals detected at the tumor site and shaved and depilated skin around the right thigh were measured and the tumor:background ratio was calculated and plotted in the graph (right panel). Soon after the initial laser exposure occurred, the signal intensity of the HS201-PDT treated tumor increased 2.5 times higher than HS201 alone at the peak while VP-PDT did not show any effect compared to VP alone. Moreover, the tumor:background ratio reached a peak at 24-h which is the most effective time for the secondary laser ablation in terms of therapeutic index. To evaluate the Hsp90 expression in the tumors in vivo, MDA-MB-231 tumors were harvested 12 h after the initial HS201 injection (6 h after the initial laser exposure) for the Western blotting analysis. Similarly to the in vitro setting, only HS201-PDT treated tumor showed increased Hsp90 protein expression compared to the others (Fig. 6c). We hypothesized that the increased Hsp90 expression in the tumor following the initial laser irradiation enhanced the accumulation of HS201 in the tumor from circulation via binding of its small molecule inhibitor structure to Hsp90 protein, which led to the rapid increase of signal intensity in the tumor site. In order to prove this, we tested a control compound HS205, which consists of VP tethered to an analog of the HS201 that cannot bind Hsp90, to compare with HS201 and VP in in vivo PDT. As a result, only HS201-PDT showed strongly enhanced signal accumulation in the tumor site after initial laser exposure of PDT, while VP-PDT had no effect and HS205-PDT showed minimum effect on signal enhancement (Supplementary Fig. 10a, b). Slightly enhanced accumulation of HS205 after HS205-PDT may be due to the enhanced permeability of tumor vasculatures caused by PDT. To confirm the enhanced accumulation of HS201 over VP in HS201-PDT or VP-PDT treated tumors, confocal microscopic analysis was performed using tumors harvested 12 h after HS201 or VP injection with/without the initial laser exposure of PDT at 6-h time point (Fig. 6d). The images of whole tumor sections are shown in Supplementary Fig. 7. In the HS201-PDT treated tumors, higher accumulation of PS in the tumor cytosol was confirmed compared to tumors from mice receiving VP-PDT or PS alone without exposure to laser light. In addition, there was a morphologic change of the plasma membrane in the HS201-PDT treated tumors, suggesting cellular degeneration caused by the treatment. These results suggest that the feedforward Hsp90 regulation enhanced the accumulation of HS201 at the tumor site after initial laser exposure but not that of VP.

### HS201-PDT significantly suppresses tumor growth of human BC

**Fig. 7:**
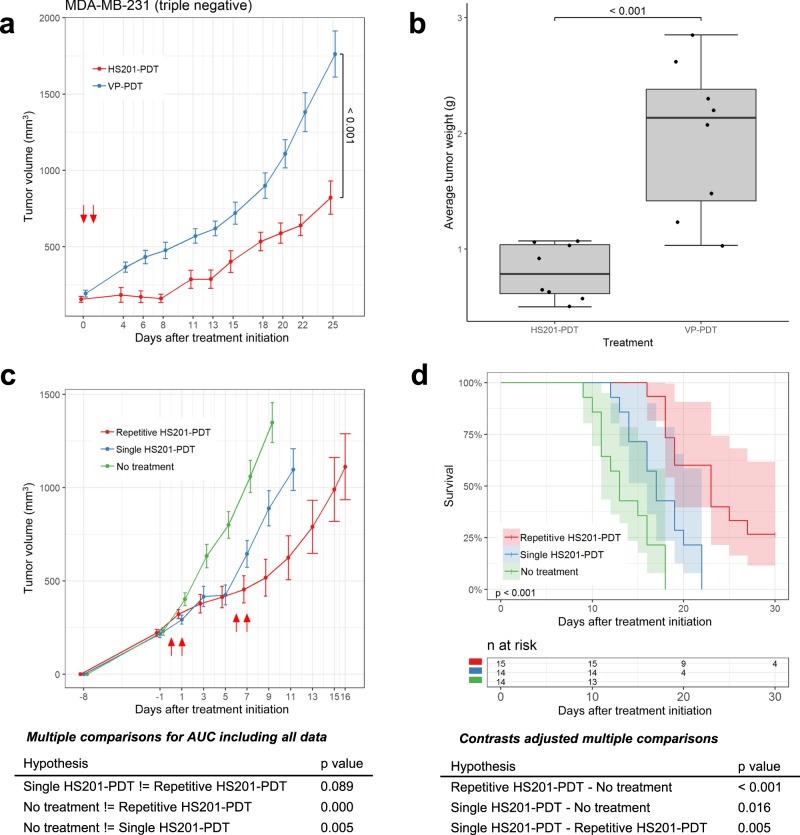
Improved antitumor effect of HS201-PDT against MDA-MB-231 tumors in SCID-beige mice. **a** Antitumor effect of HS201-PDT and VP-PDT against MDA-MB-231 tumors in vivo. MDA-MB-231 tumor-bearing mice were administered VP or HS201 (25 nmol/mouse) via tail vein. The laser irradiation (690 nm wavelength, 120 J/cm^2^/4 min) was applied to the tumor area with DLI of 6 and 24 h (red arrows). The data shown are average ± SEM of tumor volumes (*n* = 8 for each group). Student’s *t* test was performed for the comparison of two treatments. **b** Comparison of the tumor weight among treatment groups. At the end of the experiment (day 25 after treatment initiation), mice were euthanized and all tumors were excised and weighed (*n* = 8 for each group). Welch *t* test was used for statistical analysis. **c** Antitumor effect of repetitive HS201-PDT against MDA-MB-231 tumors in vivo. MDA-MB-231 tumor-bearing mice were administered VP or HS201 (25 nmol/mouse) via tail vein. The laser irradiation (690 nm wavelength, 120 J/cm^2^/4 min) was applied to the tumor area with DLI of 6 and 24 h (red arrows). Initial PDT treatment was performed on days 0 and 1 and repeated on days 6 and 7. As controls, no treatment and single HS201-PDT (treated on days 0 and 1) groups were made. Averages ± SEM of tumor volumes for each group (*n* = 14 for no treatment group, *n* = 14 for single HS201-PDT group, *n* = 15 for repetitive HS201-PDT group) are shown. ANOVA and Tukey’s test were performed for statistical analysis. Red arrows indicate laser exposures. **d** Survival of MDA-MB-231 tumor-bearing mice treated with single or repeated HS201-PDT (*n* = 14 for no treatment group, *n* = 14 for single HS201-PDT group, *n* = 15 for repetitive HS201-PDT group). Mice were counted as dead when the tumor volume reached humane endpoint according to IACUC approved protocol (>2000 mm^3^). Survival data was analyzed with the Cox Proportional Regression Model followed by Tukey’s test.

MDA-MB-231 tumor-bearing SCID-beige mice were treated with HS201-PDT or VP-PDT to compare their anti-tumor effects. Taking the previous findings into account, we optimized the treatment schedule of PDT such that laser irradiation was performed twice at 6 and 24 h after initial PS injection. In this study, the HS201-PDT group showed significant tumor growth suppression compared to the VP-PDT group (Fig. 7a). After euthanasia of the mice, all tumors were excised and weighed (Fig. 7b). The HS201-PDT treated group showed a smaller average tumor weight compared to the VP-PDT group (*p* < 0.001), supporting the tumor growth suppression by HS201-PDT.

As the MDA-MB-231 tumors treated with HS201-PDT still showed progression despite treatment, we aimed to test if repeated PDT would enhance the antitumor effect and benefit the survival of the treated mice. Repetitive HS201-PDT (on days 0 and 6) showed remarkable tumor suppressive effects after both the first and the second PDT administration (Fig. 7c). We also analyzed the survival of mice in each group (Fig. 7d). Repetitive treatment with HS201-PDT resulted in a significantly prolonged survival whereas no treatment and single HS201-PDT groups showed shorter survival durations (median survival; no treatment 13 days, single HS201-PDT 17 days, and repetitive HS201-PDT 23 days, respectively).

**Fig. 8:**
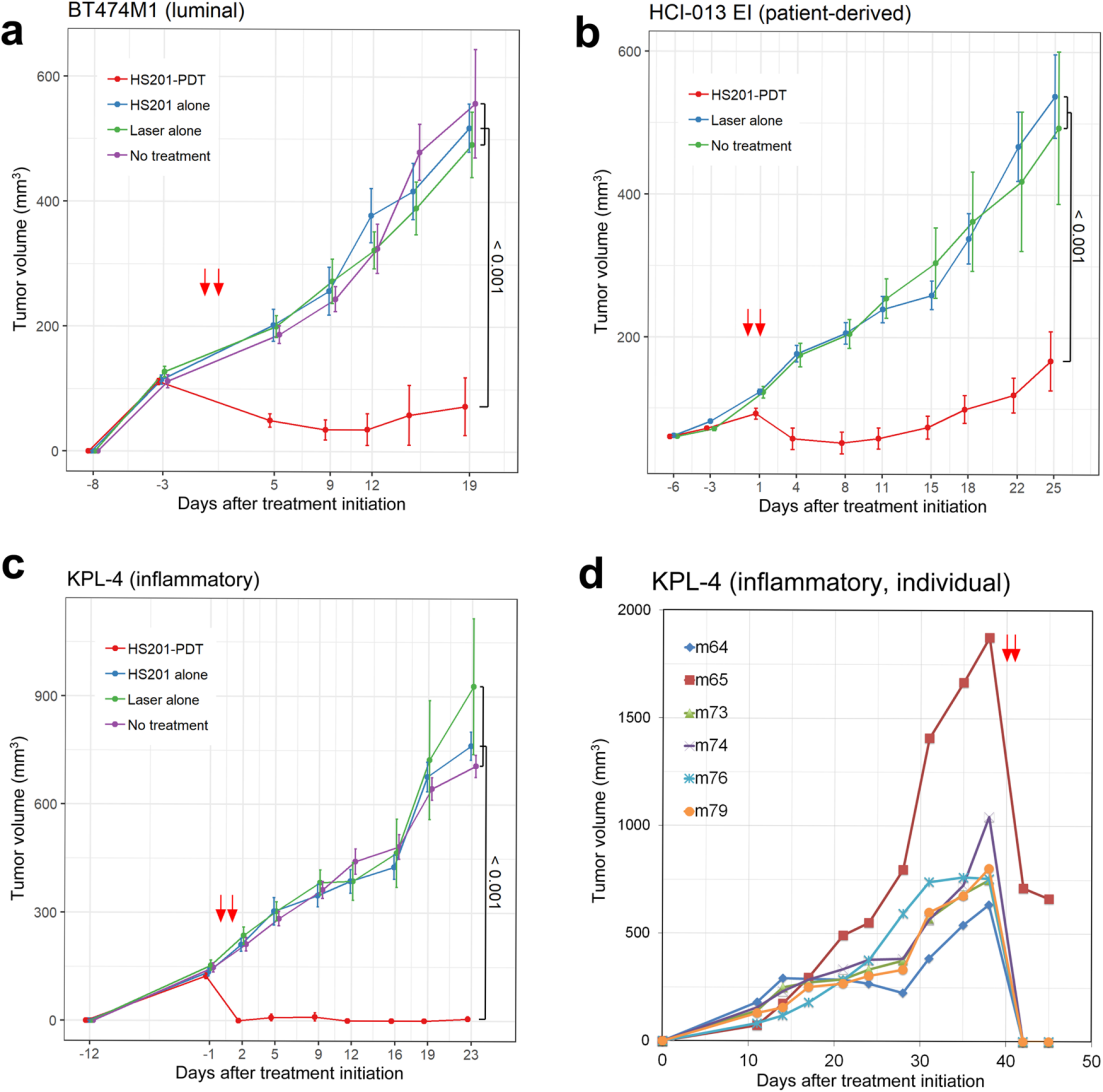
Antitumor effect of HS201-PDT against various types of human BC xenografts in SCID-beige mice. **a** Antitumor effect of HS201-PDT against BT474M1 tumors in vivo. HS201-PDT was performed against BT474M1 tumors (luminal B, ER+, HER2+) in female SCID-beige mice in the same way as indicated in Fig. 7a. As controls, no treatment group, HS201 administration alone group, and a group of laser irradiation without PS administration were made. Averages ± SEM of tumor volume for each group (*n* = 7 for no treatment group, *n* = 7 for HS201 alone group, *n* = 6 for laser alone group, and *n* = 8 for HS201-PDT group) are shown. Kruskal–Wallis test and nonparametric Dunnett’s test performed. Red arrows indicate laser exposures. **b** Antitumor Effect of HS201-PDT against patient derived HCI-013 tumors in vivo. HS201-PDT was performed against patient derived xenograft, HCI-013 tumors (invasive lobular, ER+, HER2−), as described in Fig. 7a. No treatment group and laser alone group were set as control groups. Averages ± SEM of tumor volume for each group (*n* = 8 for no treatment group, *n* = 7 for laser alone group, and *n* = 10 for HS201-PDT group) are shown. ANOVA and Dunnett’s test performed. Red arrows indicate laser exposures. **c** Antitumor effect of HS201-PDT against KPL4 tumors in vivo. HS201-PDT was performed against KPL-4 tumors (inflammatory, HER2+) as described in Fig. 7a. No treatment group, HS201 administration alone group, and laser alone group were compared. Averages ± SEM of tumor volume for each group (*n* = 8 for no treatment group, *n* = 7 for HS201 alone group, *n* = 7 for laser alone group, and *n* = 7 for HS201-PDT group, respectively) are shown. ANOVA and Dunnett’s test performed. Red arrows indicate laser exposures. **d** Antitumor Effect of HS201-PDT against KPL4 tumors at their late phase in vivo. HS201-PDT was performed against bulky KPL-4 tumors (over 600 mm^3^ in volume) on SCID-beige mice in the same way as indicated in Fig. 7a. Individual tumor volumes are shown. Red arrows indicate laser exposures.

To confirm the wide applicability of HS201-PDT to different molecular and clinical subtypes of human BC, we tested it against other human BC cell lines and patient derived xenografts implanted in SCID-beige mice. HS201-PDT had significant antitumor effects on BT474M1 (luminal B, ER+, HER2+) and the human patient derived BC xenograft, HCI-013 EI (invasive lobular, ER+, HER2−) (Fig. 8a, b). Because of the direct applicability of PDT to superficially growing BCs, we tested the treatment efficacy of HS201-PDT against 2 representative IBC lines, KPL-4 (inflammatory, HER2+) and SUM149 (inflammatory, triple negative). Importantly, HS201-PDT was effective for both KPL-4 tumors (Fig. 8c) and SUM149 tumors (Supplementary Fig. 11), and especially the response of KPL-4 tumors to HS201-PDT was dramatic even when tumor sizes approached the humane endpoint (Fig. 8d). These data demonstrate that HS201-PDT is widely applicable to different molecular and clinical subtypes of BC, induces significant tumor growth suppression, and provides survival benefit.

## Discussion

PDT is an established local ablative modality for the palliative management of symptomatic tumors. Nonetheless, current PS molecules are not tumor-selective, and therefore risk toxicity to normal tissues that are exposed to laser light and also systemic phototoxicity in response to sunlight. We previously reported the use of a nIR dye tethered to a small molecule Hsp90 inhibitor (HS131), enabling us to image aggressive breast tumors of a variety of molecular subtypes^20^. To overcome the limitations of conventional PDT, we used a similar strategy to develop an Hsp90-targeted PS and demonstrated exquisite tumor selectivity and greater anti-tumor efficacy across molecular and clinical subtypes of BC, including the notoriously aggressive IBC without significant systemic or local toxicity. This high therapeutic index allows for repeated cycles of HS201-PDT which we demonstrated to enhance survival.

Although the conventional use of targeted delivery of light by laser probes, either superficial or percutaneous, can reduce the risk of normal tissue phototoxicity, PS accumulation in tissues, such as eyes and the skin, can cause phototoxicity after exposure to daylight^29^. When using a passive PS delivery strategy for PDT, such as porfimer sodium as PS, patients experience photosensitivity periods of 6 weeks or longer due to the long elimination half-life of porfimer sodium (21.5 days). Although our strategy primarily leverages Hsp90 specific accumulation, we also employ a second-generation synthetic PS, VP, which has a shorter elimination half-life (5–6 h), leading to shorter photosensitivity periods (48 h). As a second-generation PS, VP also has intense absorption bands at longer wavelengths (690 nm), enabling treatments to greater depths. This wavelength penetration limit, rather than tumor specificity of PS provided by HS201, currently limits our treatment of deep and visceral tumors, as normal tissues in the path of laser light should be spared.

Because of the excitation and emission characteristics of HS201, our Hsp90 targeted PDT has both diagnostic and therapeutic properties. We demonstrated a greater peak accumulation in tumor cells with HS201 compared to VP, although the peak was delayed compared to a non-Hsp90 targeted photosensitizer. Our data suggest that this is because there are multiple mechanisms mediating HS201 uptake including the utilization of the LDL receptor for internalization similar to VP^26^, but the main mechanism of HS201 cellular uptake appears to be the binding to Hsp90 on the cell surface as we observed with our Hsp90 targeted imaging probes^21,30^. We speculate that differences in the size and the molecular charge of the molecule may also contribute to differences in cellular uptake. This delay in peak accumulation combined with rapid washout from normal tissue leads to a favorable tumor to background ratio, creating an excellent therapeutic index. We demonstrated that HS201 is retained in tumors longer than VP, likely due to intracellular binding of HS201 to Hsp90 protein. The longer retention provides a wider window during which laser light can be administered, while also enhancing the tumor to background ratio.

An advantageous property of the Hsp90 targeted PDT, is the feedforward mechanism. Following initial laser exposure, additional HS201 accumulation is detected in the treated tumor, thus enabling a second laser exposure with a greater therapeutic index. Weaker accumulation of non-Hsp90 targeted PSs (VP, HS205) was also observed in laser exposed tumors probably due to enhanced permeability of tumor vasculatures caused by PDT^31,32^. The same mechanism might be contributing to the enhanced accumulation of HS201 after PDT, but only to a weak level as was observed with control HS205. Our data suggest that laser treatment in conjunction with HS201 administration results in cellular stress which induces increased levels of Hsp90. Thus, additional binding of HS201 to surface Hsp90 and subsequent internalization can take place following the laser exposure. While there could be concern about increasing the levels of Hsp90 within the tumor, we observed displacement of the Hsp90 client proteins suggesting that HS201 or its metabolites may still retain Hsp90-inhibitory effects.

Although designed as a prototype, HS201 may be applicable to selected clinical situations. For example, Hsp90-targeted PDT could be used in diffuse superficial tumors, as tumor cells would be ablated, while intervening normal tissues would be spared, even after laser exposure. One such clinical scenario would be the treatment of IBC, a highly aggressive and lethal subtype^33–36^, in which malignant tumor cells are found in the dermal lymphatics^37^, rendering them within the depth of penetration of nIR wavelengths of light. We found that HS201-PDT has shown high anti-tumor efficacy against the IBC cell line KPL-4 suggesting that patients with IBC may be ideal candidates for this novel treatment modality. Due to the aggressive nature of IBC, the capacity to rapidly diagnose and treat is critical to maximizing survival. In addition, as IBC is a globally prevalent disease yet is challenging to treat in even resource rich countries, a therapeutic with the capacity for rapid implementation, such as PDT, could compliment current multimodality therapy with the potential to replace such therapy in the future.

In conclusion, we confirmed the enhanced antitumor efficacy of Hsp90 targeted PDT using a prototype molecule (HS201), which can currently be applied to tumors found up to a few cm in depth, such as IBC. In the future, other nIR delivery devices and PS excited by different wavelengths of light could be developed that would enable deeper tissue penetration. We will soon initiate a clinical trial of HS201-PDT for BC patients.

## Methods

### Cell culture

Human BC and mammary epithelial cell lines, MDA-MB-231 (triple negative), MCF-7 (luminal A, ER+, PR+, HER2−), HMEC (primary mammary epithelial cells), and MCF10A (immortalized non-tumorigenic mammary epithelial cells) were purchased from ATCC. BT474M1 (luminal B, ER+, HER2+) cell line was a more tumorigenic and metastatic subclone of BT474, and a kind gift from Dr. Dihua Yu in University of Texas MD Anderson Cancer Center (Houston, TX)^38^. KPL-4 (inflammatory, HER2+) cell line was a kind gift from Dr. Junichi Kurebayashi (Kawasaki Medical School, Japan)^39^. Patient-derived BC xenograft, HCI-013 (invasive lobular, ER+, HER2−), was kindly provided by Dr. Alana Welm while she was at the Oklahoma Medical Research Foundation (Oklahoma City, OK) and maintained in SCID mice.

MDA-MB-231, MCF-7, and KPL-4 cells were maintained in Dulbecco’s Modified Eagle’s Medium (DMEM) supplemented with 10% heat-inactivated fetal bovine serum (FBS) and penicillin/streptomycin at 37͘ °C in a humidified incubator containing 5% CO_2_. BT474M1 cells were maintained in DMEM/F12 supplemented with 10% heat-inactivated FBS and penicillin/streptomycin. MCF10A and HMEC cells were maintained in MEGM^™^ Mammary Epithelial Cell Growth Medium (Lonza, Basel, Switzerland). Cells were authenticated by morphology and growth curve analysis and were routinely screened for the mycoplasma contamination by PCR. All mycoplasma tests performed during this study showed negative.

### Reagents and antibodies

VP (Novartis Pharmaceuticals Corp., Basel, Switzerland), a well-characterized photosensitizer also serves as a nIR dye with 700 nm emission peak, and HS201, a photosensitizer made of VP tethered to an Hsp90 small molecule inhibitor (HS10), were used for in vitro and in vivo imaging and PDT. Because VP exists as two isomers, we assume the synthesis of HS201 results in two isomers. In addition, we used a control compound HS205, which consists of VP tethered to an inactive form of Hsp90 small molecule inhibitor. The compounds were developed and supplied by the Haystead Lab^21,40^ (Duke University Department of Pharmacology and Cancer Biology). See Supplementary Fig. 12a–c for the synthesis of HS201 and HS205.

Annexin V-APC, PE-CF594 Anti-CD24 antibody (clone M1/69), and FITC Anti-HER2 antibody (clone NEU 24.7) was purchased from BD Biosciences (California, USA) and 7-AAD from Beckman Coulter Inc. (California, USA). A recombinant human Hsp90α protein was purchased from Enzo Life Sciences, Inc. (New York USA). An Hsp90 inhibitor 17-AAG was purchased from MedChemExpress (New Jersey, USA). Anti-Hsp90 antibodies were purchased from Abcam plc. (clone AC88, Cambridge, UK), and Santa Cruz Biotechnology (clone F-8, California, USA). Anti-HIF1 α antibody (clone D2U3T) was from Cell Signaling Technology, Inc. (Massachusetts, USA). Anti-GAPDH antibody (clone FL-335) and anti-Akt 1/2/3 antibody (clone H-136) were from Santa Cruz. APC Anti-CD45 antibody (clone 30-F11) was purchased from BioLegend (California, USA). LIVE/DEAD Fixable Aqua Dead Cell Stain Kit was purchased from Thermo Fisher Scientific (Massachusetts, USA). Secondary antibodies (goat anti-mouse IgG, goat anti-rabbit IgG, and donkey anti-mouse IgG antibodies) conjugated with nIR dye, IRDye 800CW, were purchased from LI-COR Inc. (Nebraska, USA).

### Mice

Inbred SCID-beige mice and MMTV-neu mice were purchased from Taconic Biosciences, Inc. (New York, USA), and bred at the Duke University Cancer Center Isolation Facility. All animal studies described were approved by the Duke University Medical Center Institutional Animal Care & Use Committee and the US Army Medical Research and Materiel Command (USAMRMC) Animal Care and Use Review Office (ACURO) and performed in accordance with guidelines published by the Commission on Life Sciences of the National Research Council.

### Measurement of cellular uptake of PS in vitro

The in vitro uptake of reagents designed for nIR imaging and PDT was measured using the Odyssey CLx imaging system (LI-COR, Inc. Nebraska, USA) at 700 nm wavelength. BC cells were seeded in 96-well plates (10,000 cells/well) and cultured in each appropriate medium at 37͘ °C. After the cells reached sub- confluency in each well, VP or HS201 (0.03–10 μM, respectively) were added, incubated for 30 min, removed, and washed once with phosphate-buffered saline (PBS). The signal intensity of each well was measured at 700 nm to evaluate cellular uptake of VP and HS201.

### Confocal microscopy of cellular PS uptake in vitro

MDA-MB-231 cells were seeded on micro cover glass (VWR, Pennsylvania, USA) in 6 cm petri dishes and cultured in 10% FBS DMEM medium at 37͘ °C. Cells were separated into three exposure duration groups, 0, 3, and 6 h, to evaluate reagent retention inside the cells at each time point. When the cells reached sub-confluency in each petri dish, VP or HS201 (1.0 μM) was added, co-incubated for 30 min, removed, and washed once with PBS. For the 0-h group, cells were fixed with 10% neutral buffered formalin for 15 min at 37 °C immediately after nIR staining. As for the 3 and 6-h group, culture media were exchanged every 1, 3, or 6 times, respectively, before the cells were fixed. After the formalin was removed, the cells adherent to the micro-glass were stained with wheat germ agglutinin (WGA) Alexa Fluor 488 conjugate membrane staining dye (Invitrogen, Massachusetts, USA) and DAPI (BioLegend, California, USA) for 10 min at room temperature. After being washed, micro cover glasses were mounted on the glass slides and observed using a ZEISS LSM880 confocal microscope (Carl Zeiss AG, Oberkochen, Germany). Imaris for Cell Biologists—CL software (Bitplane, Zurich, Switzerland) was used to generate 3D images.

### Flow cytometry analysis of cellular PS uptake in vitro

MDA-MB-231 cells were seeded in 6-well plates (100,000 cells/well) and cultured in 10% FBS DMEM at 37͘ °C. After the cells reached sub-confluency in each well, VP, HS201, or HS205 (0.03–10 μM, respectively) was added, incubated for 30 min, removed, and washed once with PBS. For the 0-h group, cells were harvested and fixed with 1% neutral buffered formalin immediately after nIR staining. As for the other groups, culture media were exchanged every 1 h until the 6-h time point and every 12 h thereafter before fixation. Collected cells were acquired by an LSRII flow cytometer (BD Biosciences, California, USA). Hsp90 inhibitor blocking was performed as follows: MDA-MB-231 cells were seeded in 12-well plates (50,000 cells/well) and cultured in 10% FBS DMEM at 37͘ °C. After the cells reached sub-confluency in each well, 17-AAG (250 or 500 μM) was added, incubated for 30 min, then VP or HS201 (1 μM) was added and co-incubated for another 30 min. Cells were harvested, washed with PBS, and acquired by an LSRII flow cytometer. LDL receptor blocking was carried out as follows: MDA-MB-231 cells seeded in 6-well plates were cultured as described above until they reached sub-confluency, harvested, and suspended in serum free DMEM. Cells were co-incubated with LDL (5, 10, or 25 μM) for 1 h at 4 °C in the refrigerator and then washed with PBS. HS201 and VP (1 μM) were dissolved in serum free DMEM and incubated with the cells for 30 min at 37 °C and then washed. Collected cells were acquired by an LSRII flow cytometer.

### Killing assay

The effect of in vitro PDT using VP (VP-PDT), HS201 (HS201-PDT), and HS205 (HS205-PDT) was determined by MTT assay. BC cells seeded in a 96-well plate were co-incubated with PS and washed as described above. A laser with a wavelength of 690 nm was applied at various doses (1.88–120 J/cm^2^) to each well using ML8500 Illumination System connected to ML7710 laser machine (Modulight, Inc., Tampere, Finland). For the 0-h group, cells were irradiated with the laser immediately after nIR labeling. For the 3 and 6-h group, culture media were exchanged every 1 h for 3 or 6 times, respectively, before laser exposure. After the laser treatment, cells were cultured overnight prior to the analysis. MTT solution (20 μl) was added to each well, incubated for 2 h at 37 °C, removed, and washed by PBS once. DMSO (200 μl) was added to each well to dissolve the cells. The optical density value at 550 nm (650 nm subtracted) of each well was measured by a Model 680 Microplate Reader (Bio-Rad Laboratories, Inc., California, USA).

### Apoptosis by flow cytometry analysis

Apoptosis assays were carried out by staining cells with Annexin V-APC and 7-AAD following the addition of each reagent. Briefly, BC cells were co-incubated with HS201 or VP (1 µM final concentration) for 30 min at 37 °C, washed, resuspended in the medium, and added to 96-well plates (1.25 × 10^5^ cells/well/100 µl medium). Cells were exposed to a 690 nm laser at the indicated dose (3.75, 7.5, 15, 30 J/cm2) with irradiance of 2000 mW/cm^2^ and incubated for designated time (0, 1, 2, 4, or 24 h) at 37 °C. The control groups included cells without any treatment, cells exposed to a 690 nm laser without PS labeling, and cells labeled with PS (1 μM) alone. All the cells were resuspended in 100 μL of Annexin V binding buffer containing 5 μL of Annexin V-APC and 20 μL of 7-AAD, incubated for 15 min at room temperature, and acquired by an LSRII flow cytometer (BD Biosciences, California, USA).

Surface expression of Hsp90 was analyzed by staining cells with PE-conjugated anti-Hsp90 antibody while control cells were labeled with PE-conjugated control IgG. After 30 min incubation, cells were washed and acquired by an LSRII flow cytometer.

### Detection of Hsp90 and client proteins by Western Blot

For in vitro samples, cells were cultured on 10 cm petri dishes, treated according to each protocol, mixed with RIPA buffer in the presence of proteinase inhibitors, homogenized using a Digital Sonifier^®^ SLPe (Emerson Electric Co., Missouri, USA), and centrifuged at 13,000 rpm for 10 min at 4 °C. The supernatants were collected and aliquots were stored at −80 °C until needed. For in vivo tumor tissues, samples were collected after treatment and homogenized in RIPA buffer in the presence of proteinase inhibitors. After centrifugation at 13,000 rpm for 10 min at 4 °C, the supernatant was pooled, filtered through a 0.22 μm filter, aliquoted and stored at −80 °C until needed. Protein concentration was determined by a BCA assay. Thirty microgram of protein was applied for each lane, run on 12% Tris-HCl acrylamide gel, and transferred to polyvinylidene fluoride (PVDF) membranes. Membranes were blocked using Odyssey Blocking Buffer (LI-COR, Inc., Nebraska, USA) for 1 h at room temperature, incubated with anti-Hsp90 (dilution 1:1000), anti-HIF1α antibodies (dilution: 1:1000), anti-Akt 1/2/3 antibodies (dilution 1:1000), or anti-GAPDH antibody (dilution: 1:1000) at 4 °C overnight on the rocker. Membranes were washed with PBS or TBS with 0.05% Tween (PBS-T or TBS-T), co-incubated with IRDye 800CW Secondary Antibodies (dilution 1:5000) for 1 h at room temperature, washed with PBS-T or TBS-T, and analyzed by the Odyssey CLx imaging system (LI-COR, Inc. Nebraska USA). Band signals were quantified using Image Studio^™^ Software (LI-COR, Inc., Nebraska, USA).

### In vivo imaging

For the xenograft BC model, MDA-MB-231 cells (1 × 10^6^ cells/mouse) were injected into the right flank of female SCID-beige mice. We also used a spontaneous BC model, MMTV-neu mice. MDA-MB-231 tumor-bearing mice were administered 10 nmol of HS201 or VP via tail vein when their tumor sizes reached 10–12 mm diameter. nIR signal intensity at the tumor area was measured using the Pearl Trilogy imager (LI-COR, Inc., Nebraska, USA) at 700 nm channel over time. Two or three mice in each group were sacrificed at 24 h post-nIR dye injection to measure nIR signal accumulation in tumors and organs (lungs, liver, spleen, and kidneys). In addition, a comparison of in vivo uptake between HS201 and HS205 was done using MDA-MB-231 tumor-bearing SCID-beige mice with the same protocol using 10 nmol of PS per each mouse and measured the nIR signal intensity for a week. We also collected blood plasma from three mice in each group over time. Plasma samples were placed in 96-well plates (50 μl/well) and analyzed to evaluate nIR signal in the circulating blood. Half-life of nIR signal in tumor site and plasma was calculated using GraphPad Prism 8.0.1 (GraphPad Software, California, USA) taking the peak time point as a starting point.

### Flow cytometry analysis of cellular HS201 uptake in vivo

Tumor-bearing and non-tumor bearing MMTV-neu mice were administered 100 nmol of HS201 via tail vein. Mice were sacrificed 6 h after HS201 injection to harvest tumors and mammary gland tissues. Tumors and mammary gland tissues were digested in the media containing DNase, hyaluronidase, and collagenase for 90 min and washed to collect single cells. Cells were stained with LIVE/DEAD Fixable Aqua Dead Cell Stain Kit, APC anti-CD45 antibody, PE-CF594 anti-CD24 antibody, and FITC anti-HER2 antibody, washed, fixed, and acquired by an LSRII flow cytometer. CD45-negative and CD24-positive cells were gated as mammary epithelial-derived cells^41^. HS201-positive cells were defined according to the signal at Alexa Flour 700 channel.

### Confocal microscopy of tumor samples

VP or HS201 (500 nmol/mouse) was injected into tumor-bearing mice via tail vein as described above. Mice were sacrificed at designated time points (1, 3, 6, or 12 h) after compound injection and their tumors were harvested. A mouse without any injection was also sacrificed as a control. Tumors in some mice were irradiated with laser light (690 nm wavelength, 120 J/cm^2^/4 min) using ML7710 laser system (Modulight) at 6-h time point after the PS injection, and were collected at 12 h time point. Tumors were fixed overnight in 10% neutral buffered formalin before sectioning into 50 μm thick sections using Compresstome^TM^ Microtome. Samples were stained using WGA Alexa Fluor 488 conjugate membrane staining dye (Invitrogen) and DAPI. A ZEISS LSM880 confocal microscope (Zeiss systems, Germany) with a 40×/1.2NA oil objective was used to collect tiled images of DAPI (e.g. 405 nm/em, 410–450 nm), WGA (e.g., 488 nm/em, 490–530 nm), and VP/HS201 (e.g., 633 nm/em, 635–760 nm). Mean fluorescence intensities of the PS for the whole tumor tissue areas were calculated using FIJI software (version 2.0.0-rc-69/1.52p).

### Photodynamic therapy

BC cells (1 × 10^6^ cells/mouse) or human patient derived BC tissues were inoculated into the right flank of female SCID-beige mice. After the tumor size reached 6 mm in diameter, PDT was performed. First, VP or HS201 (25 nmol/mouse) was injected via tail vein. Six hours after the PS injection (drug-light interval (DLI) = 6 h), tumors were irradiated with a 690 nm wavelength laser at a dose of 120 J/cm^2^/4 min using the medical laser system ML7710 (Modulight). The second laser irradiation was performed at 24 h after PS injection (DLI = 24 h). In vivo imaging was performed as described above. Tumor size was monitored by caliper measurement twice a week. Tumor volume was calculated as follows; tumor volume (mm^3^) = short diameter (mm) × short diameter (mm) × long diameter (mm)/2. Mice were counted as dead when tumor volume reached humane endpoint according to IACUC approved protocol (>2000 mm^3^).

### Statistics and reproducibility

Mean and standard error were presented using spaghetti plots. Tumor volume along of time was standardized by the baseline tumor volume when baseline was different to at least one group. Areas under tumor growth curve were calculated under spline interpolation^42^ and adaptive quadrature. Continuous outcomes were compared among groups using either analysis of variance or Kruskal–Wallis test^43^ followed by parametric or nonparametric^44^ multiple comparisons. If only two groups were compared, then either Welch *t* test^45^ or Mann–Whitney^46^ test was applied. Normality assumption was verified by Shapiro–Francia^47^ test and homogeneity of variances by Levene test^48^. Survival data was compared using Log-rank test^49^ and multiple comparisons were performed using contrasts based on a Proportional Cox Model. All tests of hypotheses were two-sided and a significance level of 0.05. Calculations were performed using R, version 3.5.1^50^ or GraphPad Prism 8.0.

### Reporting summary

Further information on research design is available in the Nature Research Reporting Summary linked to this article.

## Supplementary information

Supplementary Information

Description of Additional Supplementary Files

Supplementary Data 1

Supplementary Movie 1

Reporting Summary

## Data Availability

The data that support the findings in this study are available upon reasonable request from the corresponding authors. Source data can be found in Supplementary Data 1.
