# Supplementary material for: Heat shock protein 90-targeted photodynamic therapy enables treatment of subcutaneous and visceral tumors
Source: Commun Biol. 2020 May 8;3:226. doi: 10.1038/s42003-020-0956-7 (PMC7210113; doi:10.1038/s42003-020-0956-7)
Supplement: Supplementary file 1 — Supplementary Information [file 42003_2020_956_MOESM1_ESM.pdf]

Supplementary Fig. 1

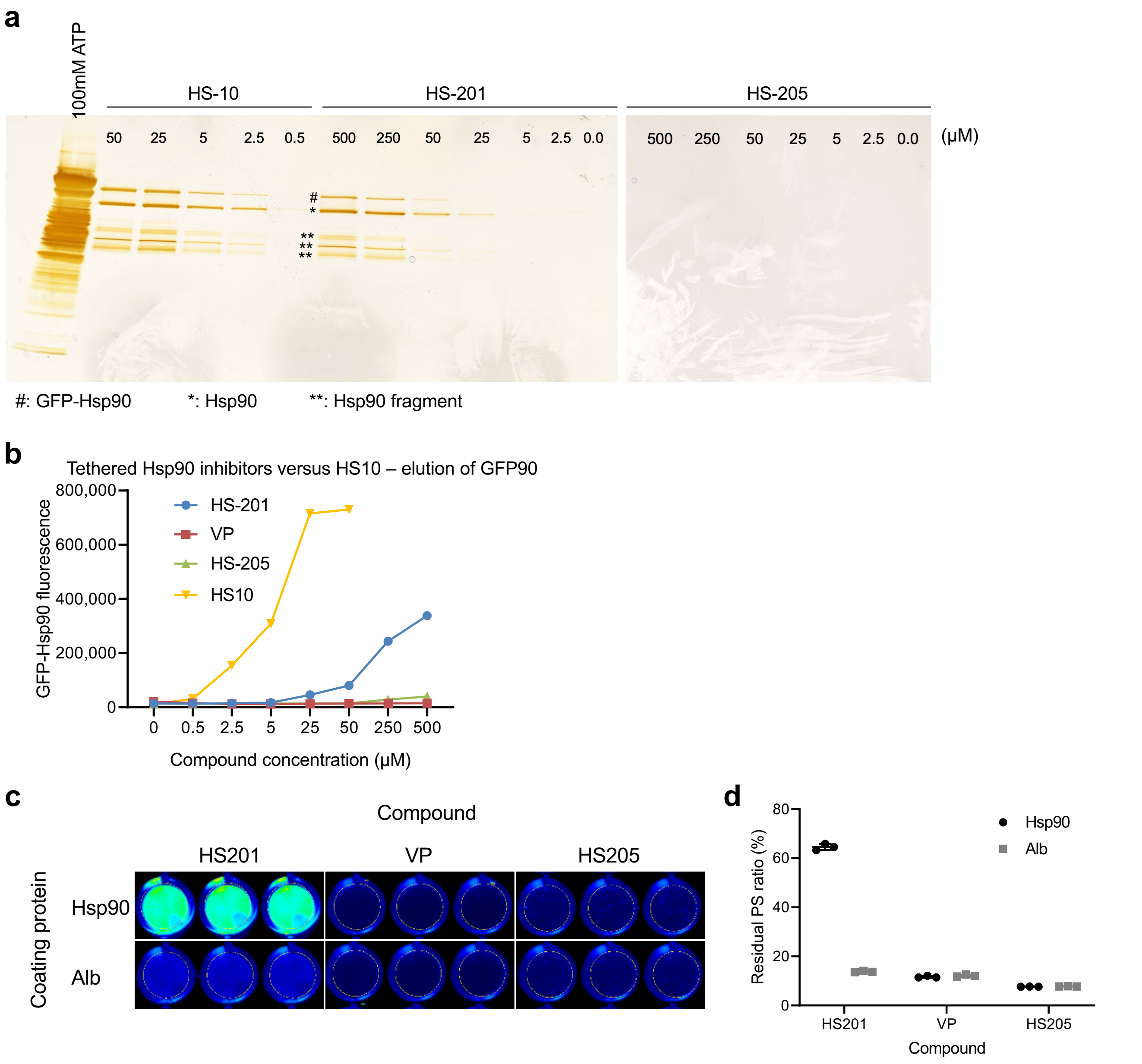

Supplementary Figure 1. HS-201 selectively binds recombinant heat shock protein 90.

a) Recombinant GFP-Hsp90a (human) was expressed in E. Coli and clarified cell extracts applied to g-phosphate linked ATP resin. Aliquots of the media (1 ml) containing the captured proteins were washed, and then eluted in parallel with the indicated molecules (HS10, HS201, HS205). The eluates were characterized by SDS-PAGE and silver staining. The indicated protein bands #: GFP-Hsp90, *: Hsp90 and **: Hsp90 fragment were identified by mass spectrometry. Note: ATP density of affinity media is ~5µmol/ml. **b)** Column eluates were measured for GFP fusion protein content by fluorescence at em488nm/em522nm. **c)** The binding activity of HS201, VP, and HS205 to purified human Hsp90 protein by ELISA. Human serum albumin was used as a control to coat the plate. The wells were filled with PSs (50 µl, 0.1 µM), incubated for one hour, and then removed using a plate washer. nIR signals were measured using a LI-COR Odyssey Imager before and after one hour incubation with PS. **d)** The ratio of residual and original nIR signals of each compound. Data are expressed as means ± SD.

Supplementary Fig. 2

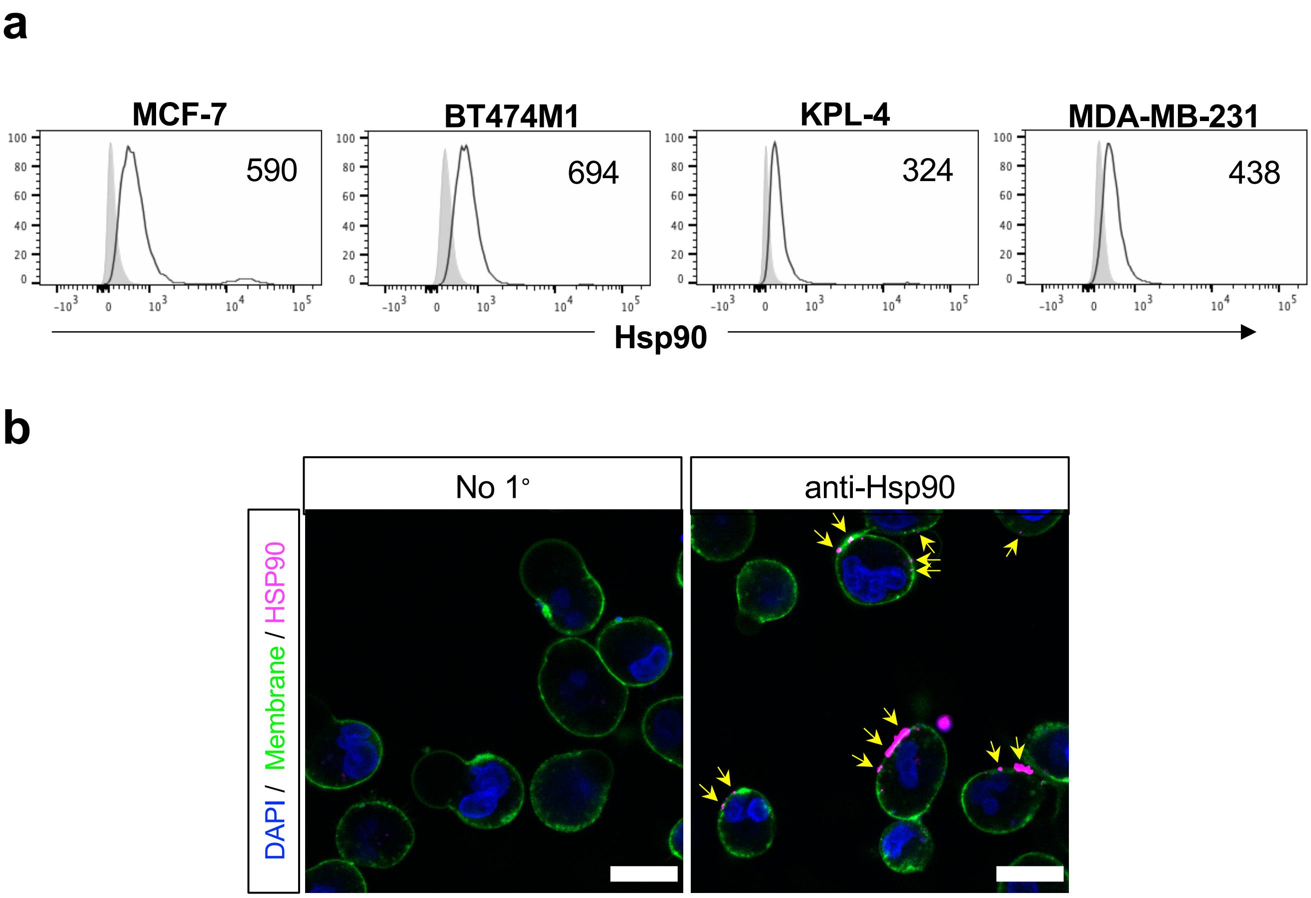

Supplementary Figure 2. Cell Surface Hsp90 Expression by Breast Cancer Cells. (**a**) Breast cancer cell lines were stained with PE-conjugated anti-Hsp90 mAb (sc13119, Santa Cruz), or control IgG for 30 min at 4C. After wash with PBS, cells were acquired by LSRII flow cytometry machine. Open histogram: anti-Hsp90, filled histogram: IgG control. Mean fluorescence intensity for Hsp90 staining is shown in each histogram. (**b**) MDA-MB-231 cells were incubated on glass bottomed dishes (MatTek) overnight, then labeled with/without anti-Hsp90 mAb (1:200 dilution, sc13119) for 30 min at 4C and then fixed. Cells were then incubated 2ndary antibody (1:500 dilution, AF568 conjugated goat anti-mouse IgG, Life Technologies), followed by WGA AF488 membrane dye (5 µg/mL, ThermoFisher) and DAPI staining. Images were acquired using Zeiss LCM880 Confocal Laser Scanning Microscope. Yellow arrows indicate cell surface labeling of Hsp90. Scale Bar = 20 µm

Supplementary Fig. 3

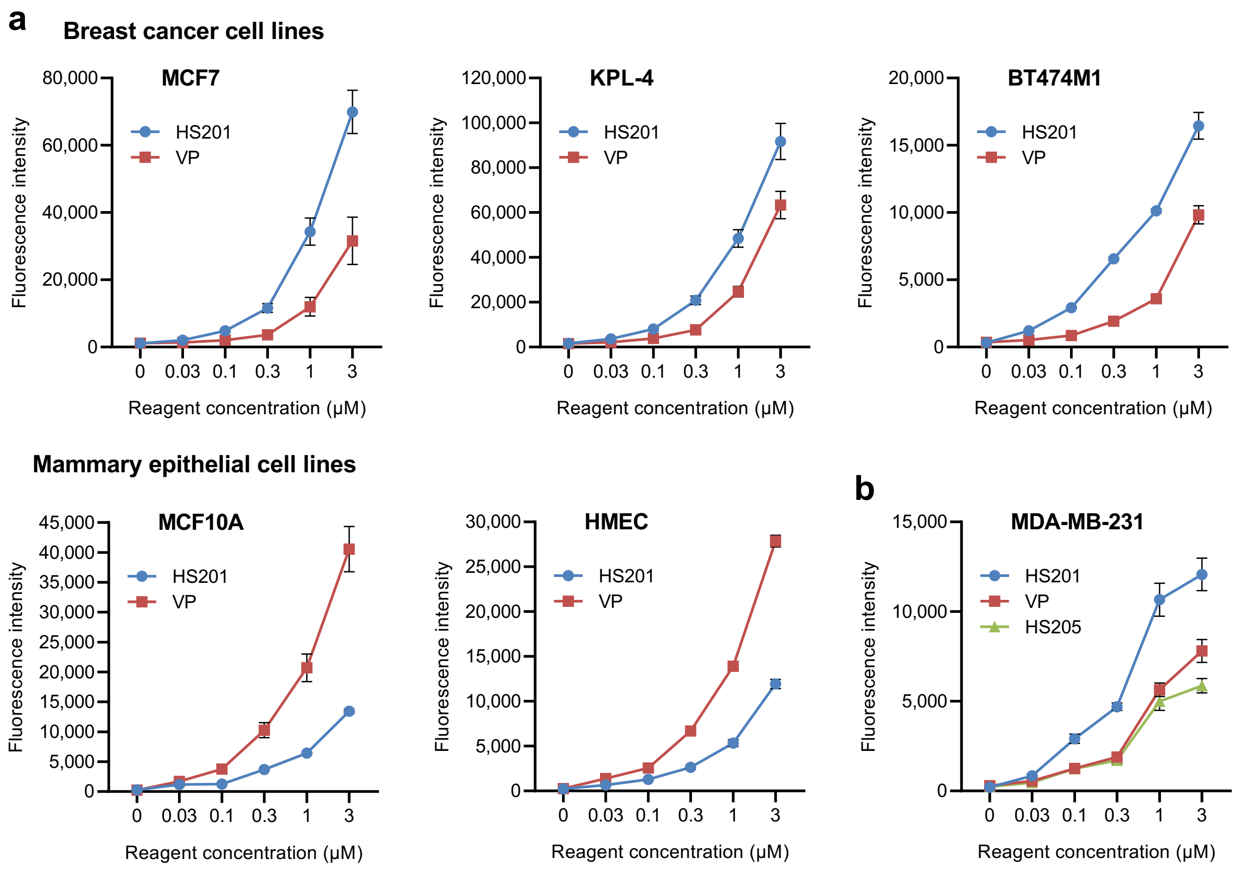

Supplementary Figure 3. Uptake of HS201, VP, and HS205 by BC cells and mammary epithelial cells *in vitro*. a) Uptake of HS201 and VP by BC cells and mammary epithelial cells *in vitro*. Human BC cells (MCF-7, KPL-4, and BT474M1) and mammary epithelial cells (MCF-10A and HMEC) seeded in 96-well plates were labeled with HS201 or VP (0 - 3 μM, respectively) for 30 minutes at 37℃, removed, and washed by PBS once. nIR fluorescence intensity of each well was measured at 700nm by an Odyssey CLx imaging system. Data are expressed as means ± SD. **b)** Uptake of HS201, VP, and HS205 by MDA-MB-231 cells *in vitro*. MDA-MB-231 cells seeded in 96-well plates were labeled with HS201, VP, or HS205 (0 -3 μM, respectively) for 30 minutes at 37℃, removed, and washed by PBS once. nIR fluorescence intensity of each well was measured at 700nm by an Odyssey CLx imaging system. Data are expressed as means ± SD.

Supplementary Fig. 4

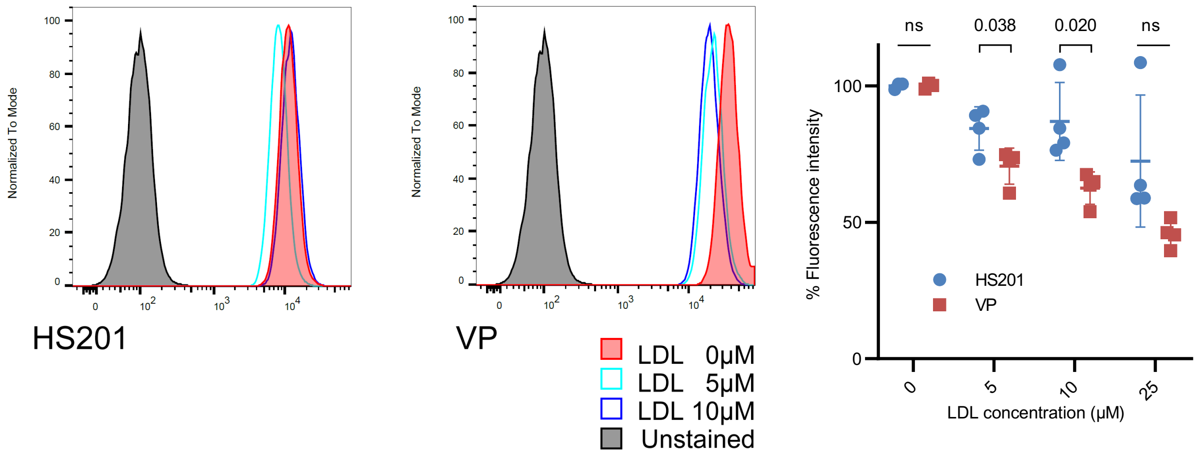

Supplementary Figure 4. LDL receptor blockade inhibits the cellular uptake of HS201 and VP. MDA-MB-231 cells were suspended in serum free DMEM, pre-treated by LDL (0, 5, 10 or 25 μM) for 1 hour at 4℃, and washed by PBS once. Then the cells were incubated with PS (HS201 or VP, 1μM) for 30 minutes at 37℃, then removed, and washed by PBS once. Fluorescence intensity was analyzed by an LSRII flow cytometer, and the representative case for each condition is shown in the histograms. Mean fluorescence intensities of the samples are shown as the percentages to the control condition (no LDL pre-treatment) in the dot plot. N=3 samples for LDL 0 μM condition, N=4 samples for 5, 10 and 25 μM conditions. Student's t-test was performed for statistical analysis. Data are expressed as means ± SD.

Supplementary Fig. 5

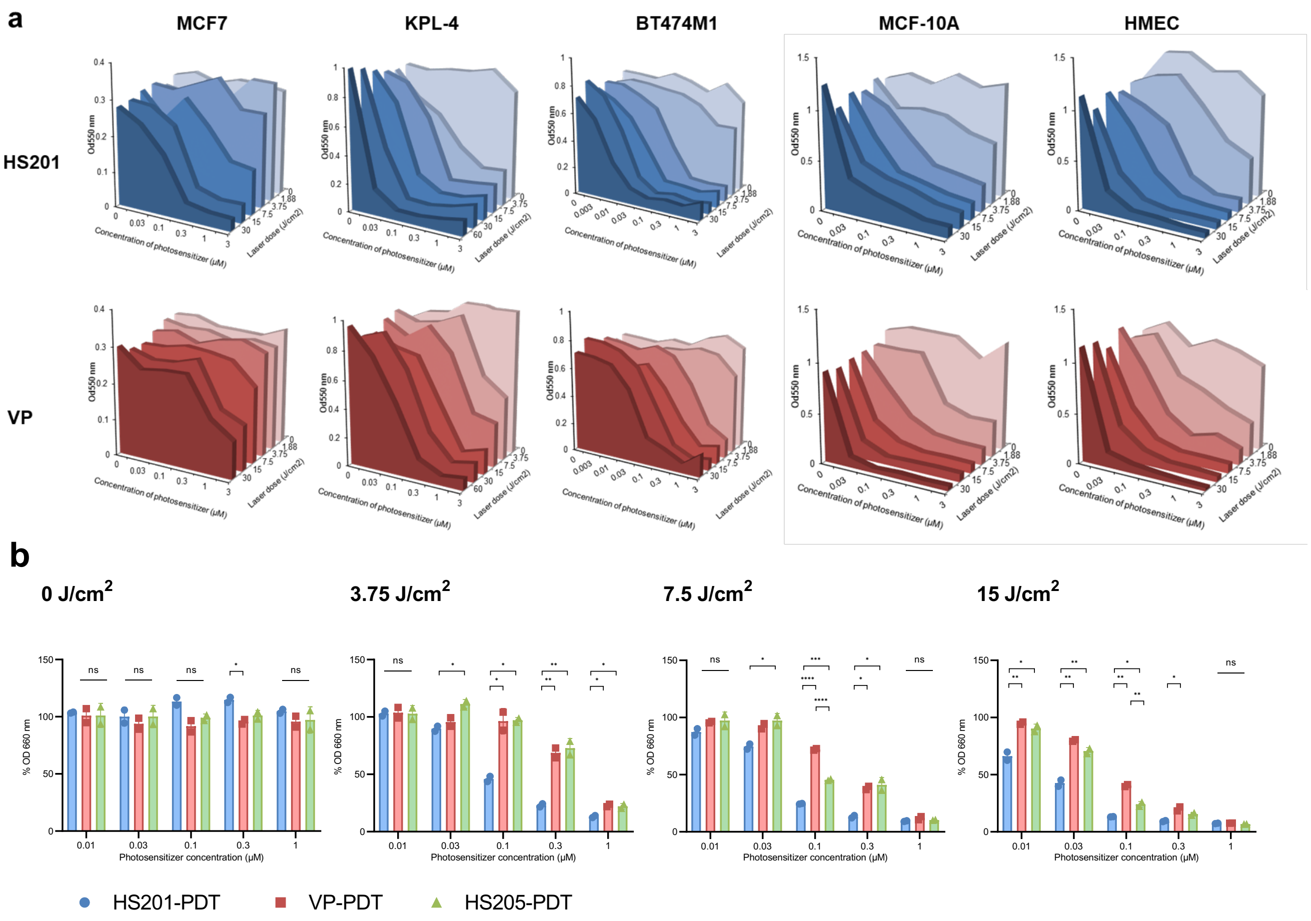

Supplementary Figure 5. Cytotoxicity of *in vitro* HS201-PDT, VP-PDT, and HS205-PDT in human BC lines and mammary epithelial cells. a) Cytotoxicity of *in vitro* PDT: Comparison of HS201 and VP in human BC lines and mammary epithelial cells. Human BC cells (MCF-7, KPL-4, and BT474M1) or mammary epithelial cells (MCF-10A and HMEC) seeded in 96-well plates were labeled with HS201 or VP as indicated (0 - 3 μM) for 30 minutes at 37℃, removed, washed by PBS once, and immediately irradiated with 690 nm wavelength laser (0 - 30 J/cm^2). Viability of the cells was analyzed by MTT assay. Upper graphs show the results for HS201-labeled cells, and lower graphs show VP-labeled cells. **b)** Cytotoxicity of *in vitro* PDT: Comparison of HS201, VP, and HS205 in MDA-MB-231 cells. MDA-MB-231 cells seeded in 96-well plates were labeled with HS201, VP, or HS205 (0 – 1 μM) for 30 minutes at 37℃, removed, washed by PBS once, and immediately irradiated by 690 nm wavelength laser (0 - 15 J/cm^2). Viability of the cells was analyzed by MTT assay. The graph shows percentages of OD value when compared with the unlabeled samples with PS. Data are expressed as means ± SD. One-way ANOVA and Tukey's multiple comparison test were performed for statistical analysis. P values: $^{*}p < 0.05$, $^{**}p < 0.01$, $^{***}p < 0.001$, $^{****}p < 0.0001$.

Supplementary Fig. 6

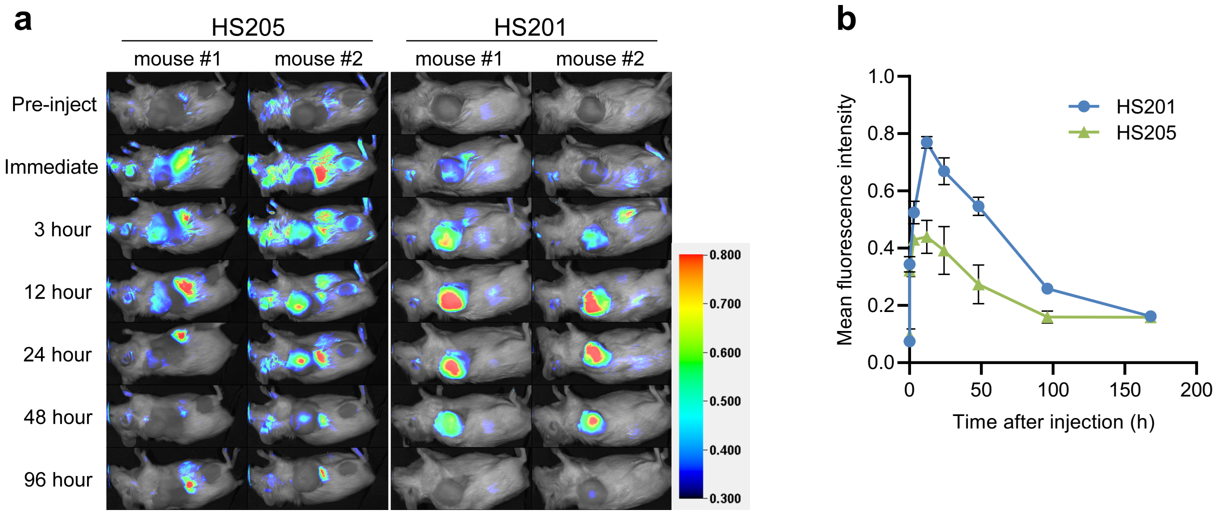

Supplementary Figure 6. Temporal dynamics of HS201 and HS205 distribution in human BC xenograft-bearing mice. a) Temporal dynamics of nIR signal from tumor area in MDA-MB-231 tumor-bearing mice injected with HS201 or HS205. MDA-MB-231 tumor-bearing mice (10 ~12 mm diameter) were administered with 10 nmol of HS201 or HS205 via tail vein. Whole body images of the mice and nIR signal intensities from tumor areas were detected by the Pearl Trilogy imaging system at 700 nm channel over time (pre-injection, immediate, 3, 12, 24, 48, and 96 hours after injection). **b)** Temporal dynamics of nIR signal from MDA-MB-231 tumors by in vivo imaging. Fluorescence intensities were monitored for individual tumors over time by Pearl Trilogy imaging system, and plotted in the graphs. Data are expressed as means ± SEM (n=3, respectively).

Supplementary Fig. 7

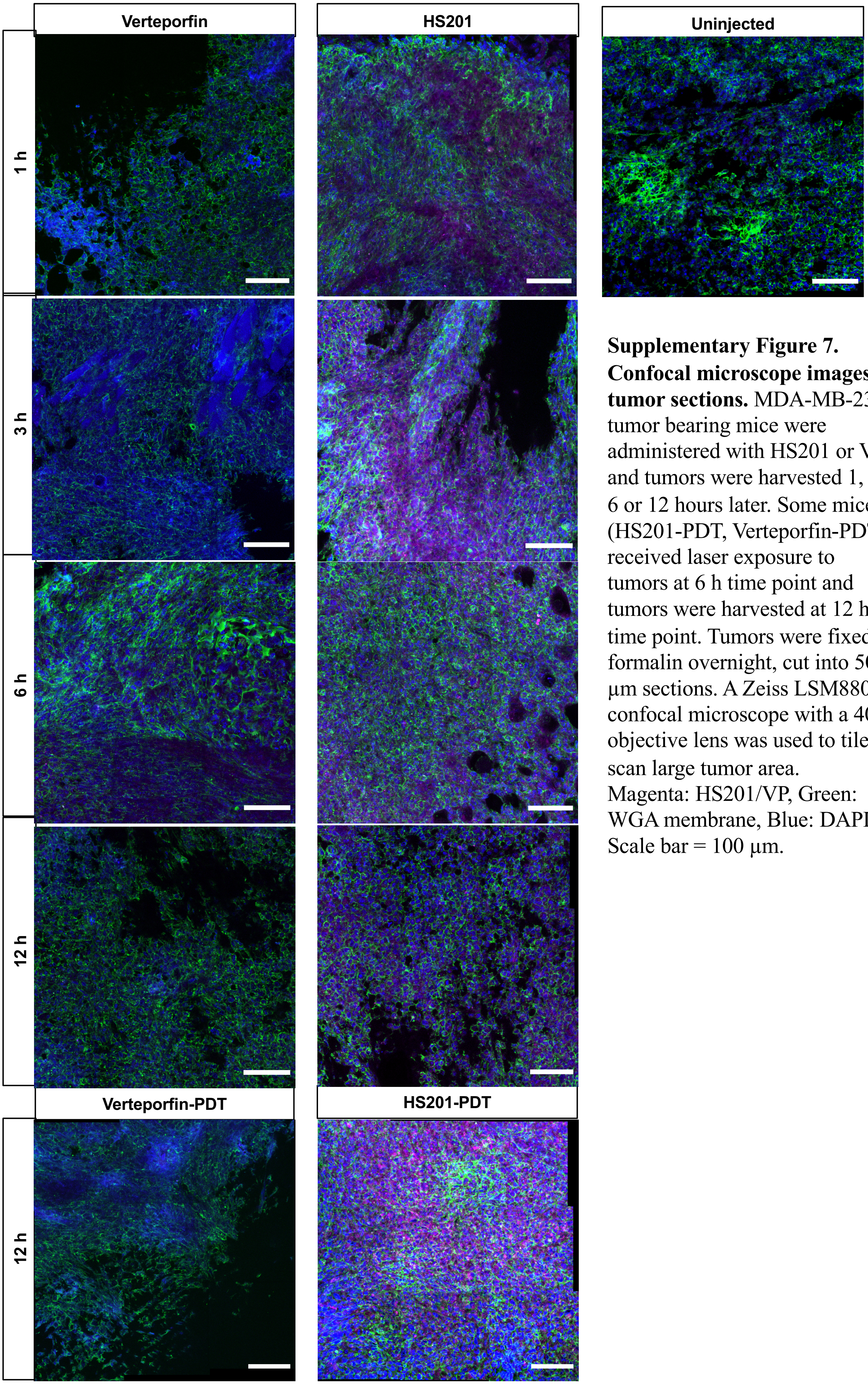

Supplementary Figure 7. Confocal microscope images of tumor sections. MDA-MB-231 tumor bearing mice were administered with HS201 or VP, and tumors were harvested 1, 3, 6 or 12 hours later. Some mice (HS201-PDT, Verteporfin-PDT) received laser exposure to tumors at 6 h time point and tumors were harvested at 12 h time point. Tumors were fixed in formalin overnight, cut into 50 µm sections. A Zeiss LSM880 confocal microscope with a 40x objective lens was used to tile scan large tumor area. Magenta: HS201/VP, Green: WGA membrane, Blue: DAPI. Scale bar = 100 µm.

Supplementary Fig. 8

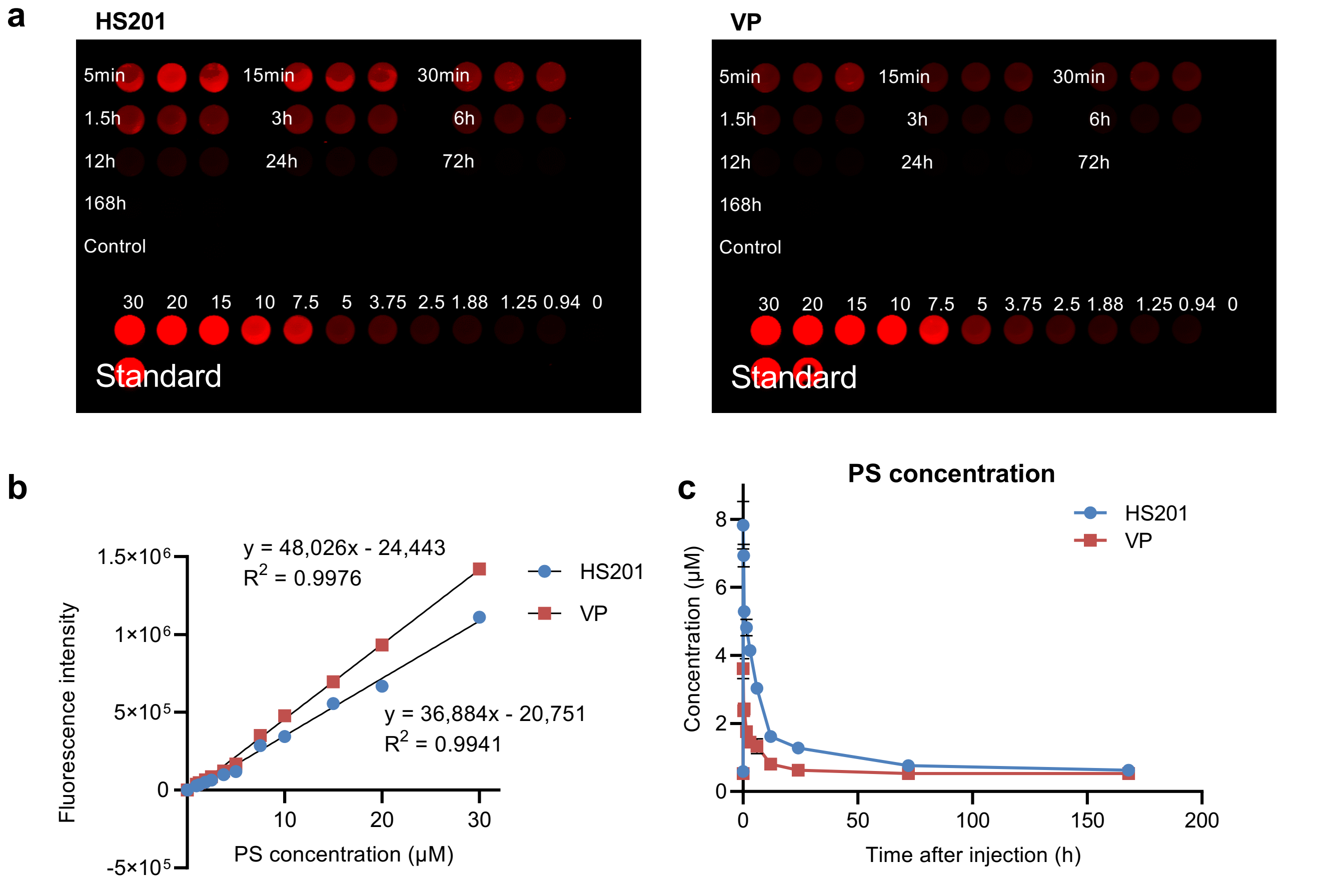

Supplementary Figure 8. Temporal dynamics of nIR signal level in the blood plasma of SCID-beige mice after HS201 or VP injection. a) nIR signal at 700 nm wavelength emitted from PS (HS201 and VP) diluted in murine blood plasma. Non-tumor bearing SCID-beige mice were injected with HS201 or VP (1mg/kg) and sacrificed at each designated time point to collect blood plasma. Blood plasma was added (50 µl/well) to each well of 96-well flat-bottomed plate (n=3 for each time point). In addition, indicated amount of PS (HS201 or VP, 0 – 30 µM) was added to each well of the plate to generate regression lines. nIR signal intensity was measured at 700nm by an Odyssey CLx imaging system using 700 nm channel. **b)** Regression line calculated from fluorescence intensity of PS (HS201 and VP). Fluorescence intensity of HS201 and VP diluted in murine blood plasma (0 - 30µM) was measured using a LI-COR Odyssey Imager and regression line was calculated. **c)** Temporal dynamics of nIR signal in murine blood plasma after intravenous injection of PS (HS201 or VP). PS concentration in blood plasma was calculated based on the regression line. Half-life of HS201 and VP was 3.3 and 1.7 hours, respectively. Data are expressed as means ± SEM (n=3, respectively).

Supplementary Fig. 9

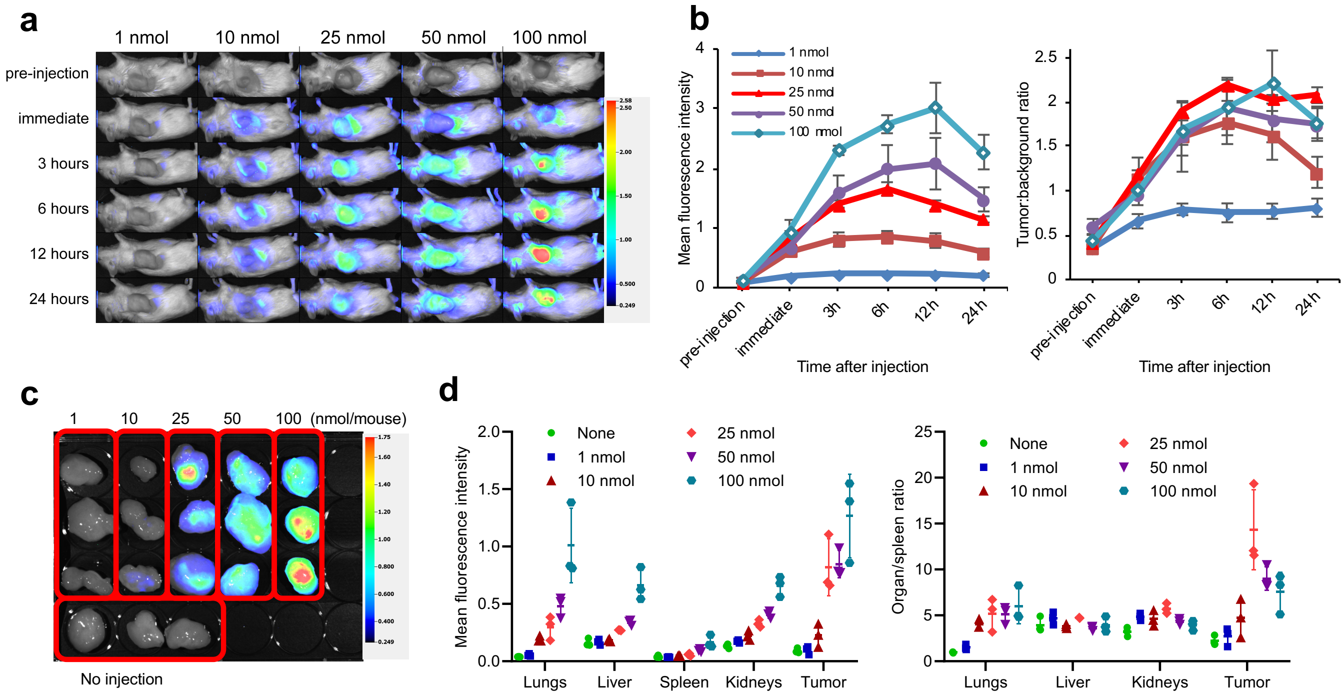

Supplementary Figure 9. Comparison among various doses of HS201 in distribution to organs and tumors in human BC xenograft-bearing mice. a) Comparison of nIR signal from MDA-MB-231 tumors in mice administrated with various HS201 dosages. MDA-MB-231 tumor-bearing mice (10 ~12 mm diameter) were administered with 1-100 nmol of HS201 via tail vein. nIR signals from tumor area were detected using a Pearl Trilogy imaging system at 700 nm channel over time (pre-injection, immediate, 3, 6, 12, and 24 hours after injection). Three mice were assigned to each HS201 dosage group and administered HS201 as described above. Representative mice from each dosage group are shown. **b)** Temporal dynamics of nIR signal from MDA-MB-231 tumors in vivo comparing various HS201 dosages. MDA-MB-231 tumor-bearing mice were administered with 1-100 nmol of HS201. Left panel: Fluorescence intensities were monitored for individual tumors over time by a Pearl Trilogy imaging system, and plotted in the graphs. Right panel: The ratios of nIR signal detected in tumor site and background skin around ear were also calculated and plotted as tumor-background ratio. Data are expressed as means ± SEM (n=3). **c)** Ex vivo imaging of excised MDA-MB-231 tumors comparing various HS201 dosages. MDA-MB-231 tumor-bearing mice administered with 1-100 nmol of HS201 were sacrificed 24 hours after PS injection and tumors and organs were collected. Images of nIR signal distribution in the tumors were analyzed by a Pearl Trilogy imaging system. **d)** Ex vivo detection of nIR signal of excised tumors and organs. Left panel: Mean fluorescence intensities of nIR signals from each organs and tumors are shown. Right panel: The nIR signal ratios of each organ or tumor to spleen are also shown. Data are expressed as means ± SEM.

Supplementary Fig. 10

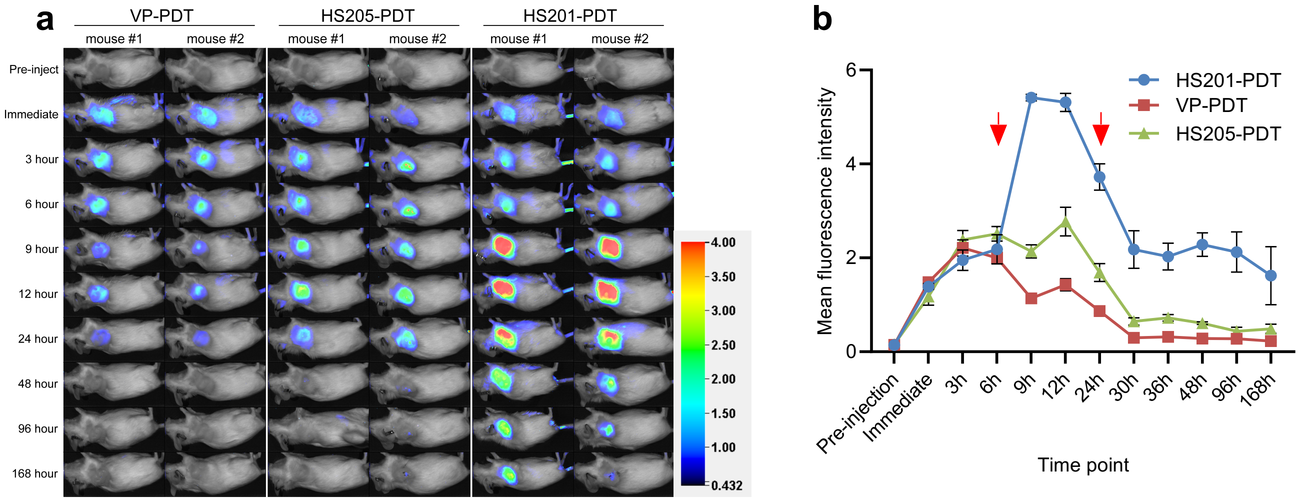

Supplementary Figure 10. Enhanced HS201 accumulation in human BC xenografts after Laser exposure. a) Whole body images of MDA-MB-231 tumor-bearing mice treated by HS201-PDT, HS205-PDT, or VP-PDT. HS201, HS205 (PS conjugated with altered Hsp90 inhibitor) or VP (25 nmol/mouse) were administered to MDA-MB-231 tumor-bearing mice, and tumors were irradiated with laser (690 nm, 120 J/cm2/4min) at 6 and 24-hour time points. Whole body images were taken over time (pre-injection, immediate, 3, 6, 12, 24, 48, 96, and 168 hours after injection) by a Pearl Trilogy imaging system at 700 nm channel, and representative 2 mice from each group are shown. **b)** Temporal dynamics of nIR signal intensity of MDA-MB-231 tumors showing enhanced accumulation of HS201 but not VP or HS205 after Laser exposure. Averages ± SEM of nIR signal intensity acquired from 3 mice for each group are plotted in the graph. Red arrows indicate the timing of laser irradiation.

Supplementary Fig. 11

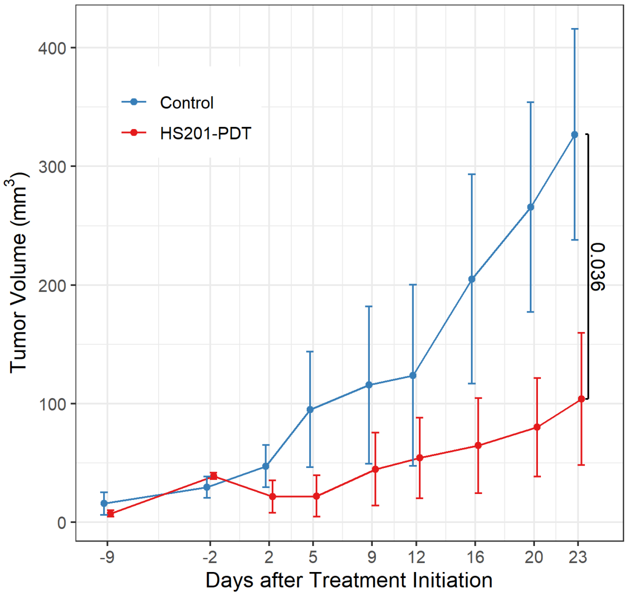

Supplementary Figure 11. Antitumor Effect of HS201-PDT against SUM149 Tumors *in vivo*. SUM149 tumor-bearing SCID mice were injected with HS201 (25 nmol/mouse) via tail vein. Six and 24 h later, laser light (690 nm wavelength) was exposed to tumors at the dose of 120 J/cm^2 each time in HS201-PDT treatment group (n=5). Mice in the control group received no treatment (n=3). Tumor size was measured twice a week. Average tumor volume is presented as means ± SEM. Mann-Whitney test was performed for statistical analysis. Two mice out of 5 in HS201-PDT group showed eradication of tumors while all mice in control group had growing tumors.

Supplementary Fig. 12

a

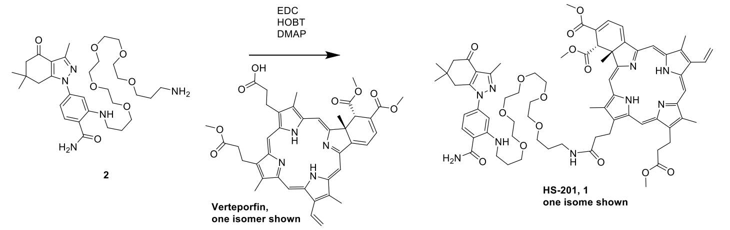

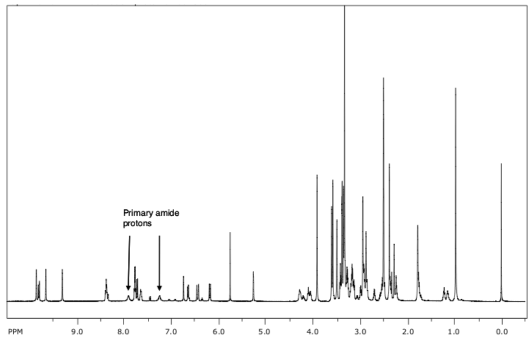

b

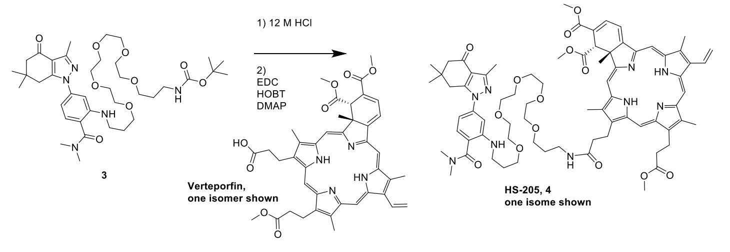

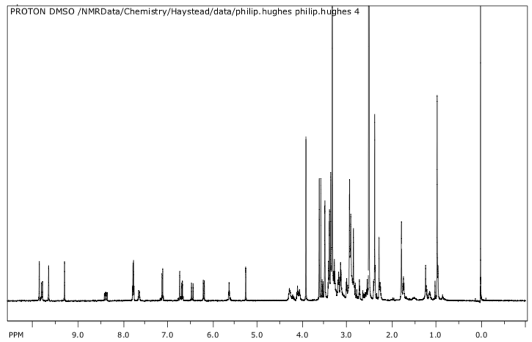

c

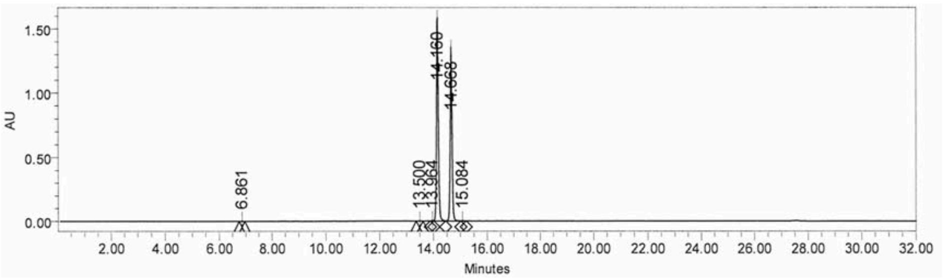

Supplementary Figure 12. Synthesis of HS201 and HS205. Reagents were obtained from commercial sources and used without further purification. Proton NMR spectra were obtained on Varian 400 and 500 MHz spectrometers. LC/MS were obtained on an Agilent ion-trap LC/MS system. Chromatography was performed using an ISCO CombiFlash Rf+ instrument. **a)** HS201. A methylene chloride solution (2 mL) of amine **2** [1] (109 mg, 180 µmol) was added to Verteporfin (MedChem Express, 100 mg, 139 µmol), HOBT (21 mg, 139 µmol), DMAP (3 mg) and EDC (53 mg, 278 µmol) followed by Hunig's base (36 mg, 278 µmol) and stirred to dissolve everything. After 2 h, the entire mixture was added to a column and chromatographed (silica gel, 0 to 10% MeOH in CH_2Cl_2) to give **1** (171 mg, 92%) as a brown dark crunchy solid. TLC (9/1: CH_2Cl_2/MeOH) gave a single spot with $R_f = 0.52$. LC/MS (C-18) gave two peaks with m/z= 652.8 $[M + 2H]^{2+}$. ^{1}H-NMR is consistent with the structure. **b)** HS205. Dimethylamide BOC amide **3** [2] (41.2 mg, 56 µmol) was dissolved in methylene chloride and treated with 12 M HCl (5 drops). The sample was sonicated. After 30 m, TLC showed complete BOC removal. The mixture made homogeneous with a few drops of methanol and concentrated. The residue was then treated with Verteporfin (MedChem Express, 36 mg, 51 µmol), HOBT (8 mg, 51 µmol), DMAP (2 mg) and EDC (20 mg, 101 µmol), dissolved in methylene chloride (2 mL), treated with Hunig's base (~200 µL) and stirred for 16 h. The entire mixture was added to a column (12 g isco silica) and chromatographed (2 x 12 g isco, 0 to 10% MeOH in CH_2Cl_2) to give **4** (66 mg, 97%) as a hard brown glass. LC/MS gave two peaks with m/z = 666.7 for $[M + 2H]^{2+}$ and 1332.2 for $[M + H]^{+}$. ^{1}H-NMR is consistent with the structure. **c)** HPLC analysis (Waters XBridge C18 Column (3.5 mM, 4.6 Å~ 150 mm, 2 to 98% acetonitrile in water, both with 0.025% TFA, UV at 260 nm) of the final compound HS201 gave two well separated peaks with a total integration of 99.6%.

Supplementary Fig. 13

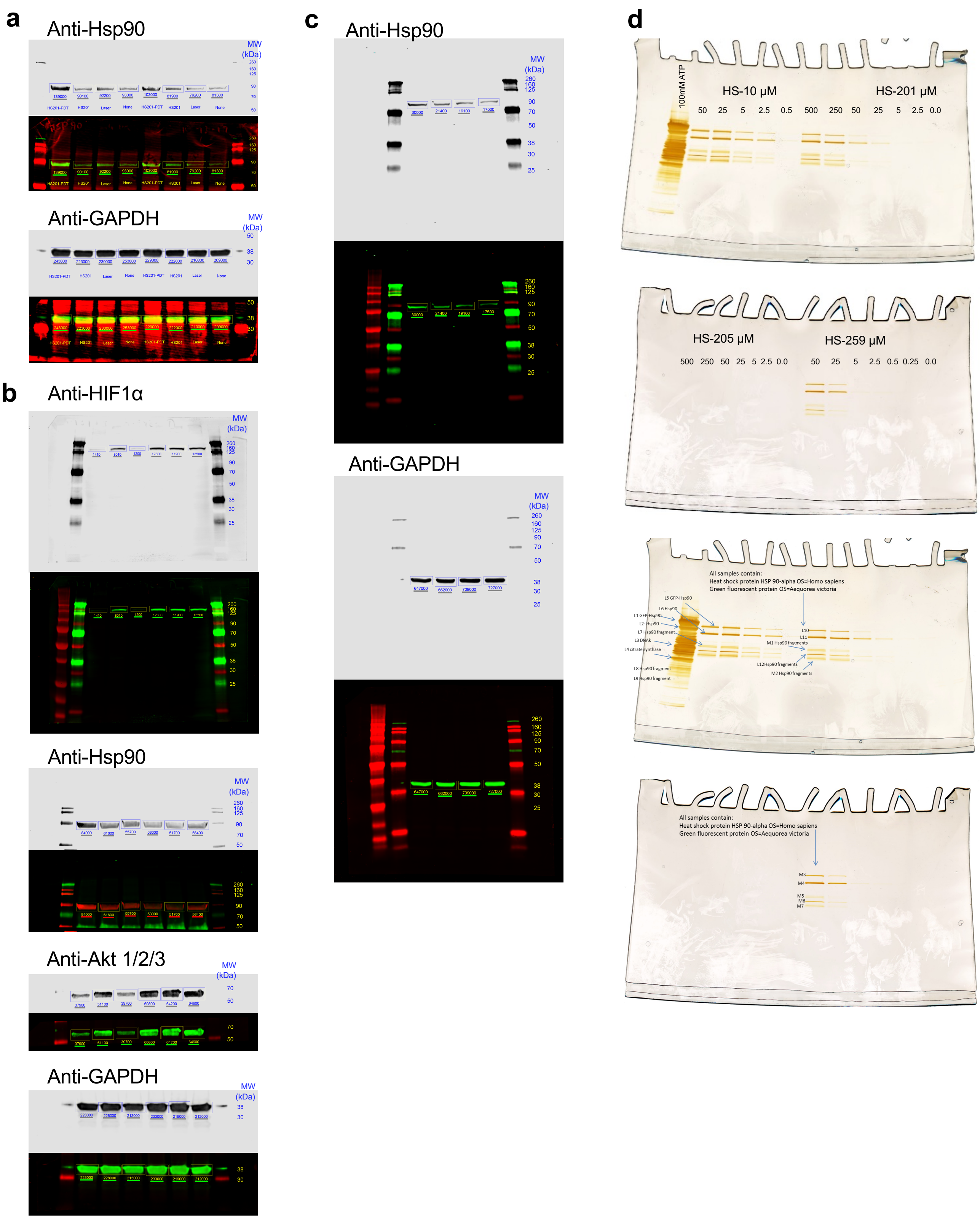

Supplementary Figure 13. Full-length blots and gels. **a)** Full-length blots from Fig. 5a. Black & white images and nIR florescence images analyzed by an Odyssey CLx imaging system are shown (red: 700nm, green; 800nm). **b)** Full-length blots from Fig. 5c. **c)** Full-length blots from Fig. 6c. **d)** Full-length gels from Supplementary Fig. 1.

Supplementary Movie 1.

3D image of HS201 uptake in MDA-MB-231 tumor cells. MDA-MB-231 cells were co-incubated with HS201 (1 μM), and then stained with WGA Alexa Fluor 488 conjugate membrane staining dye and DAPI as described in Fig. 1c. Sample slides were observed by a ZEISS LSM880 confocal microscope. Original magnification: Objective 63×. 3D data was generated by Imaris for Cell Biologists – CL software. Red: HS201, Green: WGA membrane staining, Blue: DAPI.

Supplementary References

1. Hughes, P. F., *et al*. *Bioorganic & Medicinal Chemistry* **20**, 3298-3305 (2012).
2. Crowe, L. B., *et al*. *ACS Chem Biol* **12**, 1047-1055 (2017).
