# Supplementary material for: Heat shock protein 90-targeted photodynamic therapy enables treatment of subcutaneous and visceral tumors
Source: Commun Biol. 2020 May 8;3:226. doi: 10.1038/s42003-020-0956-7 (PMC7210113; doi:10.1038/s42003-020-0956-7)
Supplement: Supplementary file 2 — Description of Additional Supplementary Files [file 42003_2020_956_MOESM2_ESM.pdf]

## Description of Additional Supplementary Files

**File Name**: **Supplementary Movie 1**
**Description**: The movie shows cytoplasmic localization of HS201 by 3 dimensional images. It demonstrates the uptake of HS201 into cytoplasm by breast cancer cells in vitro.

**File Name**: **Supplementary Data 1**
**Description**: It contains the raw data obtained in this research and used for the making of main Figures 1-8. 2)
