# Supplementary material for: Heat shock protein 90-targeted photodynamic therapy enables treatment of subcutaneous and visceral tumors
Source: Commun Biol. 2020 May 8;3:226. doi: 10.1038/s42003-020-0956-7 (PMC7210113; doi:10.1038/s42003-020-0956-7)
Supplement: Supplementary file 5 — Reporting Summary [file 42003_2020_956_MOESM5_ESM.pdf]

nature research

Corresponding author(s): H. Kim Lyerly, Timothy AJ. Haystead

Last updated by author(s): Apr 6, 2020

Reporting Summary

Nature Research wishes to improve the reproducibility of the work that we publish. This form provides structure for consistency and transparency in reporting. For further information on Nature Research policies, see Authors & Referees and the Editorial Policy Checklist.

Statistics

For all statistical analyses, confirm that the following items are present in the figure legend, table legend, main text, or Methods section.

n/a	Confirmed	
☐	☒	The exact sample size (*n*) for each experimental group/condition, given as a discrete number and unit of measurement
☐	☒	A statement on whether measurements were taken from distinct samples or whether the same sample was measured repeatedly
☐	☒	The statistical test(s) used AND whether they are one- or two-sided<br>*Only common tests should be described solely by name; describe more complex techniques in the Methods section.*
☒	☐	A description of all covariates tested
☐	☒	A description of any assumptions or corrections, such as tests of normality and adjustment for multiple comparisons
☐	☒	A full description of the statistical parameters including central tendency (e.g. means) or other basic estimates (e.g. regression coefficient) AND variation (e.g. standard deviation) or associated estimates of uncertainty (e.g. confidence intervals)
☐	☒	For null hypothesis testing, the test statistic (e.g. *F*, *t*, *r*) with confidence intervals, effect sizes, degrees of freedom and *P* value noted<br>*Give P values as exact values whenever suitable.*
☒	☐	For Bayesian analysis, information on the choice of priors and Markov chain Monte Carlo settings
☒	☐	For hierarchical and complex designs, identification of the appropriate level for tests and full reporting of outcomes
☒	☐	Estimates of effect sizes (e.g. Cohen's *d*, Pearson's *r*), indicating how they were calculated

Our web collection on statistics for biologists contains articles on many of the points above.

Software and code

Policy information about availability of computer code

Data collection	No customized software was used. BD FACSDiva 6.1.3 was used to collect flow cytometry data. The confocal microscope images were obtained using Zeiss ZEN 2.3 SP1 (black edition). nIR fluorescence intensity data in vitro and in vivo and western blot data were collected by Li-COR Image Studio ver. 1.0.11. Additional information about software was described in the manuscript or available upon request.
Data analysis	No customized software was used. Statistical analyses were performed using R3.5.1 or Graphpad Prism 8.0.1. The confocal microscope images were analyzed using Zeiss ZEN 2.3 (blue edition) and 3D image was generated using Bitplane Imaris for Cell Biologists. Mean fluorescence intensities of the PS for the whole tumor tissue areas were calculated using FIJI software (version 2.0.0-rc-69/1.52p). Flow cytometry data were analyzed using Flowjo 10.4.2.

For manuscripts utilizing custom algorithms or software that are central to the research but not yet described in published literature, software must be made available to editors/reviewers. We strongly encourage code deposition in a community repository (e.g. GitHub). See the Nature Research guidelines for submitting code & software for further information.

Data

Policy information about availability of data

All manuscripts must include a data availability statement. This statement should provide the following information, where applicable:

- Accession codes, unique identifiers, or web links for publicly available datasets
- A list of figures that have associated raw data
- A description of any restrictions on data availability

The data that support the findings in this study are available upon reasonable request from the corresponding authors.

Field-specific reporting

Please select the one below that is the best fit for your research. If you are not sure, read the appropriate sections before making your selection.

☒ Life sciences ☐ Behavioural & social sciences ☐ Ecological, evolutionary & environmental sciences

For a reference copy of the document with all sections, see nature.com/documents/nr-reporting-summary-flat.pdf

Life sciences study design

All studies must disclose on these points even when the disclosure is negative.

Sample size	No statistical method was used to predetermine the sample size. Sample sizes for animal studies were based on previous experience that showed significance. For imaging studies, animals or samples were at least used in triplicate. For tumor growth and survival studies, at least 6 animals for each group were used to obtain informative results.
Data exclusions	No data were excluded for all figures.
Replication	All the experiments were conducted at least twice and could be reliably reproduced.
Randomization	Animals in tumor studies were randomized into groups equalizing mean tumor size at the start of the treatment.
Blinding	The investigators were not blinded to study to account for appropriate handling during the PDT procedure. The investigators were blinded to the group allocation during the tumor size measurement and nIR imaging process.

Reporting for specific materials, systems and methods

We require information from authors about some types of materials, experimental systems and methods used in many studies. Here, indicate whether each material, system or method listed is relevant to your study. If you are not sure if a list item applies to your research, read the appropriate section before selecting a response.

Materials & experimental systems

n/a	Involved in the study	
☐	☒	Antibodies
☐	☒	Eukaryotic cell lines
☒	☐	Palaeontology
☐	☒	Animals and other organisms
☒	☐	Human research participants
☒	☐	Clinical data

Methods

n/a	Involved in the study	
☒	☐	ChIP-seq
☐	☒	Flow cytometry
☒	☐	MRI-based neuroimaging

Antibodies

Antibodies used

Antibodies used for flow cytometry.
PE anti-Hsp90 antibody (abcam, Cat# ab65171, clone AC88, 1:100)
PE-CF594 anti-CD24 antibody (BD Biosciences, Cat# 562477, clone M1/69, 1:400)
FITC anti-HER2 antibody (BD Biosciences, Cat# 340553, clone NEU 24.7, 1:100)
APC anti-CD45 antibody (BioLegend, Cat# 103112, clone 30-F11, 1:400)
Anti-Hsp90 antibody (Santa Cruz Biotechnology, clone F-8, 1:200)

Antibodies used for western blot.
Anti-Hsp90 antibody (abcam, Cat# ab13492, clone AC88, 1:1000)
Anti-HIF1 α antibody (Cell Signaling, Cat# 14179S, clone D2U3T, 1:1000)
Anti-GAPDH antibody (Santa Cruz, Cat# sc-47724, clone 0411, 1:1000)
Anti-Akt 1/2/3 antibody (Santa Cruz, Cat# sc-8312, clone H-136, 1:1000)
IRDye 800CW Goat anti-Rabbit IgG Secondary Antibody, (Li-COR, Cat# 926-32211, 1:5000)
IRDye 800CW Goat anti-Mouse IgG Secondary Antibody, (Li-COR, Cat# 926-32210, 1:5000)
IRDye 680RD Donkey anti-Mouse IgG Secondary Antibody (Li-COR, Cat# 926-68072, 1:5000)
IRDye 680RD Donkey anti-Rabbit IgG Secondary Antibody (Li-COR, Cat# 926-68073, 1:5000)

Validation

Antibodies for flow cytometry were validated by titration using known negative and positive cell line. For Western blots, antibodies were validated by including positive control lysates.

Eukaryotic cell lines

Policy information about cell lines

Cell line source(s)	MDA-MB-231, MCF-7, and HMEC were purchased from ATCC.<br>BT474M1, a tumorigenic and metastatic subclone of BT474, was obtained from University of Texas MD Anderson Cancer Center (Houston, TX).<br>KPL-4 was obtained from Kawasaki Medical School, Japan.<br>HCI-013, a patient-derived breast cancer xenograft, was obtained from Oklahoma Medical Research Foundation (Oklahoma City, OK).
Authentication	The cell lines were previously characterized as referenced in the manuscript and used without further authentication.
Mycoplasma contamination	All cell lines tested negative for mycoplasma contamination.
Commonly misidentified lines<br>(See ICLAC register)	No commonly misidentified cell lines were used.

Animals and other organisms

Policy information about studies involving animals; ARRIVE guidelines recommended for reporting animal research

Laboratory animals	Female SCID-beige mice aged 6-8 weeks and female MMTV-neu mice aged 7 months or later were used.
Wild animals	The study did not involve wild animals.
Field-collected samples	The study did not involve field-collected samples.
Ethics oversight	All animal experiments were performed in accordance with a protocol approved by the Duke University Medical Center Institutional Animal Care & Use Committee and the US Army Medical Research and Materiel Command (USAMRMC) Animal Care and Use Review Office (ACURO) and performed in accordance with guidelines published by the Commission on Life Sciences of the National Research Council.

Note that full information on the approval of the study protocol must also be provided in the manuscript.

Flow Cytometry

Plots

Confirm that:

☒ The axis labels state the marker and fluorochrome used (e.g. CD4-FITC).

☒ The axis scales are clearly visible. Include numbers along axes only for bottom left plot of group (a 'group' is an analysis of identical markers).

☒ All plots are contour plots with outliers or pseudocolor plots.

☒ A numerical value for number of cells or percentage (with statistics) is provided.

Methodology

Sample preparation	For the in vitro detection of nIR signal emitted from photosensitizer, cultured cells were incubated with photosensitizer for 30 minutes, removed, and washed with PBS before being fixed with 1% neutral buffered formalin. Collected cells were acquired by an LSRII flow cytometer (BD Biosciences, California USA). LDL receptor blocking was carried out as follows: MDA-MB-231 cells seeded in 6-well plates were cultured as described above until they reached sub-confluency, harvested, and suspended in serum free DMEM. Cells were co-incubated with LDL (10 or 25 µM) for 1 hour at 4°C in the refrigerator and then washed with PBS. HS201 and VP (1 µM) were dissolved in serum free DMEM and incubated with the cells for 30 minutes at 37°C and then washed. Collected cells were acquired by an LSRII flow cytometer.<br>For the apoptosis assay, treated cells were resuspended in 100 µL of Annexin V binding buffer containing 5 µL of Annexin V-APC and 20 µL of 7-AAD, incubated for 15 minutes at room temperature, and acquired by an LSRII flow cytometer.<br>For the analysis of surface expression of Hsp90, cells were stained with PE-conjugated anti-Hsp90 antibody while control cells were labeled with PE-conjugated control IgG. After 30 min incubation, cells were washed and acquired by an LSRII flow cytometer.<br>For the in vivo HS201 uptake assay, tumor-bearing and non-tumor bearing MMTV-neu mice were administered 100 nmol of HS201 via tail vein. Mice were sacrificed six hours after HS201 injection to harvest tumors and mammary gland tissues. Tumors and mammary gland tissues were digested in the media containing DNase, hyaluronidase, and collagenase for 90 minutes and washed to collect single cells. Cells were stained with LIVE/DEAD Fixable Aqua Dead Cell Stain Kit, APC anti-CD45 antibody, PE-CF594 anti-CD24 antibody, and FITC anti-HER2 antibody, washed, fixed and acquired by an LSRII flow cytometer. CD45 negative and CD24 positive cells were gated as mammary epithelial-derived cells. HS201 positive cells were defined according to the signal at Alexa Flour 700 channel.
Instrument	LSR II flow cytometer (BD Biosciences) was used to collect flow cytometry data.

Software	BD FACSDiva 6.1.3 and FlowJo 10.4.2 were used to collect and analyze the flow cytometry data, respectively.
Cell population abundance	For analysis, at least 20,000 total events were acquired for all FACS analysis.
Gating strategy	All cells were gated based on forward and side-scatter characteristics to limit debris. Cells were gated based on positive and fluorescence minus one controls, and the frequencies of cells staining positive for each marker was recorded.

☐ Tick this box to confirm that a figure exemplifying the gating strategy is provided in the Supplementary Information.
